# Quantifying the dynamic wing morphing of hovering hummingbird

**DOI:** 10.1098/rsos.170307

**Published:** 2017-09-20

**Authors:** Masateru Maeda, Toshiyuki Nakata, Ikuo Kitamura, Hiroto Tanaka, Hao Liu

**Affiliations:** 1 School of Engineering, Tokyo Institute of Technology, 2-12-1 Ookayama, Meguro-ku, Tokyo 152-8550, Japan; 2 Graduate School of Engineering, Chiba University, 1-33 Yayoi-cho, Inage-ku, Chiba 263-8522, Japan; 3 Shanghai Jiao Tong University and Chiba University International Cooperative Research Center, Chiba University, 1-33 Yayoi-cho, Inage-ku, Chiba 263-8522, Japan; 4 Yamaha Motor Co., Ltd, 3078 Arai, Arai-cho, Kosai, Shizuoka 431-0302, Japan

**Keywords:** bird flight, feather, hovering, hummingbird, wing morphing, 3D shape reconstruction

## Abstract

Animal wings are lightweight and flexible; hence, during flapping flight their shapes change. It has been known that such dynamic wing morphing reduces aerodynamic cost in insects, but the consequences in vertebrate flyers, particularly birds, are not well understood. We have developed a method to reconstruct a three-dimensional wing model of a bird from the wing outline and the feather shafts (rachides). The morphological and kinematic parameters can be obtained using the wing model, and the numerical or mechanical simulations may also be carried out. To test the effectiveness of the method, we recorded the hovering flight of a hummingbird (*Amazilia amazilia*) using high-speed cameras and reconstructed the right wing. The wing shape varied substantially within a stroke cycle. Specifically, the maximum and minimum wing areas differed by 18%, presumably due to feather sliding; the wing was bent near the wrist joint, towards the upward direction and opposite to the stroke direction; positive upward camber and the ‘washout’ twist (monotonic decrease in the angle of incidence from the proximal to distal wing) were observed during both half-strokes; the spanwise distribution of the twist was uniform during downstroke, but an abrupt increase near the wrist joint was found during upstroke.

## 1. Introduction

Hummingbirds (family Trochilidae) are the only bird group that is capable of sustained hovering and are reported to have the highest mass-specific metabolic rate among vertebrates [1]. Some species of hummingbirds migrate between North America and Central America. To help manage such high energy demands, the wing shape and the wing kinematics (the way in which they flap wings) are likely to be sophisticated, among other traits. This includes reductions in the inertial power and in the aerodynamic power. To reduce the inertial power, the mass of the wing should be decreased, or the mass of the wing should be concentrated to the proximal portion of the wing (i.e. close to the wing base). One of the unique features of the hummingbird wing is that the arm wing is very short and the hand wing is relatively long, and the joint between them (elbow joint) cannot be stretched because of the anatomical restriction [2,3]. This feature results in more than half the wing length (distance between wing base and wing tip) of a hummingbird’s wing being composed solely of primary feathers, hence the mass is concentrated to the proximal portion and the inertial power would be reduced. Consequently, hummingbirds cannot flex (fold) their wings during upstroke as other birds do. The extent of flexing is measured by the span ratio, which is defined as the wingspan at mid-upstroke divided by the wingspan at mid-downstroke. Here, a wingspan is the distance between the left and right wing tips, and it varies with time when an animal flaps its wings. The span ratio of rufous hummingbirds is nearly 100% at hovering and never less than 85% at higher flight speeds, whereas other birds have span ratios between 20 and 80% (see [4], fig. 10).

Rather than flexing, a hummingbird wing is rotated around its long axis during supination (transition from downstroke to upstroke) and then kept outstretched during upstroke. Because of this wing rotation around its long axis (hereafter, ‘torsional reversal’), the upstroke of a hummingbird is aerodynamically active, i.e. generating useful forces (lift and/or thrust). Specifically, during hovering, approximately one-quarter to one-third of the weight support (upward component of the aerodynamic force supporting the weight of the bird) is generated during upstroke [5,6], which is considerably larger than that of other birds. For example, pied flycatchers are known to generate negligible weight support during upstroke [7], although recent studies have shown that birds in slow flight can generate forward force (thrust) during upstroke [8,9] (but not weight support).

The no wing flex during upstroke and the aerodynamically active upstroke in a hovering hummingbird are in fact more similar to the features of insect flight than to those of larger birds (for aerodynamically active upstroke in hovering insects, see e.g. [10]). Insect wing aerodynamics has been intensively studied [11] following the finding of the novel unsteady aerodynamic force generation mechanism called leading-edge vortex (LEV) [12]. Many of the insect flight studies assume the wing to be a rigid flat plate, with no wing deformation and its kinematics is described by the three degrees of freedom (d.f.) angular motion around the wing base. Although there are several pioneering works [13,14], the study on dynamic wing morphing is rather new, and only recently have precise measurements via stereo high-speed digital video recordings and three-dimensional reconstruction been performed, partly because such measurements require more than one high-speed camera, which are quite expensive. A minimum of two cameras are required to obtain the three-dimensional rigid body motion via stereo camera calibration, and when an object deforms, three or more cameras are generally necessary (but note the single camera can be used with a light grid [15]). The development of computational fluid dynamics (CFD), a numerical simulation technique to obtain the flow field around the animal, is also important because even if one has the dynamic wing morphing data, it is very difficult to precisely reproduce such dynamic shape changes mechanically. Using CFD, one can compute the three-dimensional flow with realistic wing morphing and obtain the resulting aerodynamic force and aerodynamic power. With these techniques, it has been shown that insects do exhibit time-varying changes in wing shape during flapping flight, and that these dynamic morphings are helpful in efficient aerodynamic force generation [16–18]. Generally, in-flight wing shapes of vertebrates are still largely unknown, with a few exceptions, particularly for bats [19–22].

The three-dimensional shapes of birds’ wings have rarely been described. The pioneering works by Bilo [23,24] described the wing twist and camber of a house sparrow. Liu *et al*. [25] described the wing geometry and kinematics for several bird species, but the conditions of the wings were unclear. The wing surface reconstruction of the landing steppe eagle by Carruthers *et al.* [26] is probably the first of such attempts on a live, flying bird [26,27]. The eagle was just about to land on a perch; thus, the wing was almost stationary (not flapping). Moreover, only the arm wing was reconstructed. The complete wing shape during flapping was recently obtained from a barn owl in forward flight [28], and more recently the wing and body upper surface of the parrotlet was obtained using projected line patterns [15]. The aerodynamic benefit of the bird wing morphing is not well understood, particularly for flapping flight. Note, however, that there is an interesting example of how wing morphing is related to aerodynamics in gliding flight: the adjustment of the sweep angle in the wing of a swift, which is related to the surface roughness and boundary layer flow over the wing [29–31].

We have developed a new method for reconstructing a bird’s wing surface using the visually observable natural features, mainly the wing outline and feather shafts (rachides). This method has a few advantages over the other methods. Our method is purely non-invasive, i.e. requires only the camera images and does not require any physical interference with the bird. The direct contact for placing the tracking points or markers on the animal surface is unnecessary, and projecting the light pattern such as dots or lines to the bird is not required; therefore, the alteration of the birds behaviour would be minimized. Furthermore, the simple ‘camera only’ system allows us to use it either in a wind tunnel, an aviary or potentially in the wild. Another advantage of the current method is that we not only obtain the bulk shape of the wing, but also the anatomically meaningful information, i.e. the feather shafts. For example, the sliding of the adjacent feathers during flight can be assessed. Here, we apply this technique to the wing of a hovering hummingbird to demonstrate its effectiveness.

For hummingbirds, wingbeat frequency and wingbeat amplitude were often the only kinematic parameters measured to help interpret the energy expenditure from more direct measures, such as oxygen consumption measurements [32]. For example, during the late 1990s to the early 2000s, Dudley and his colleagues worked on acquiring data on the effect of oxygen and/or air density on hovering performance. In their 2003 paper, Altshuler & Dudley noted that ‘little is known about modulation of detailed wingbeat kinematics, including such features as angle of attack (AoA), torsion along the wing, wing rotational velocities, and temporal changes in wing area related to positional changes of the feathers’ [33]. Tobalske *et al*. [4] made a step towards this direction by obtaining the three-dimensional kinematics of a few points on the wing for rufous hummingbirds across ranges of flight speeds, where they quantified many important kinematic parameters, including stroke plane angle, positional angle, span ratio (from wing span), angle of incidence and effective AoA, among others [4]. Remarkably, they found that the span ratio was slightly less than 1.0, i.e. the wing is in fact slightly flexed during upstroke, particularly during fast forward flight, indicating that the wing is capable of morphing to some degree. Recently, the existence of camber [5,34,35] and spanwise twist [6] have been reported, and the deformation within the two-dimensional camera image was also reported [36]. However, the wing shape data were usually limited to the wing outlines and the explicit quantities of the three-dimensional dynamic wing morphing, such as variation in wing area, amount of spanwise bending, spanwise twist or camber, have not been described.

To quantify the details of the three-dimensional wing shape of a hummingbird during hovering, we recorded the flight using four digital high-speed video cameras and reconstructed a three-dimensional wing model. During the reconstruction, we tracked individual feather shafts because most of the hummingbird’s wing surface is composed of flight feathers, particularly primary feathers (hummingbirds have ten primary feathers and only six or seven secondary feathers for each wing). Over a single wing stroke, substantial variations in the wing area, spanwise bending, camber and spanwise twist were observed and quantified. The potential causes and benefits of these wing morphings are discussed. A direct assessment of the aerodynamic impact of the wing morphing is underway using CFD and will be presented elsewhere.

## 2. Methods

### 2.1. High-speed video recording

The hovering flight of an amazilia hummingbird (*Amazilia amazilia*) was recorded in the Tama Zoological Park in Tokyo, Japan, on 10 November 2012 (electronic supplementary materials, videos S1–S4). The hummingbird lived in a large greenhouse that is approximately 1140 m^2^ in area and 16 m in ceiling height. The sex and exact age of the recorded individual are unknown, but it was at least 10 years old. There were three amazilia hummingbirds in the greenhouse, but each had its own territory, and we believe that the same individual always came to the same feeder. The air temperature was approximately 22^°^C. Four high-speed digital video cameras, three FASTCAM SA3 and a FASTCAM SA2 (Photron Ltd., Japan), were mounted on tripods and placed around a feeder where the bird frequently visited to hover-feed the nectar (Nektar-Plus, NEKTON GmbH, Germany). The image resolutions were 1024×1024 pixels for SA3 and 2048×1080 pixels for SA2. The cameras were synchronized via BNC cables at a frame rate of 2000 frames per second. The exposure time (shutter speed) for each camera was set at 1/3000 s. A smaller exposure time would have been preferable to reduce motion blur, but we were only allowed to use natural ambient light. A white background made of paperboards was placed to improve the contrast of the bird’s outline. The closest distance between the paperboard and the left wing tip was estimated to be approximately 100 mm, which is approximately 1.4 times the wing length and can have a minor aerodynamic interference to the wing. However, for this study we neglected the potential wall effect and we assumed that the wing kinematics and morphing were bilaterally symmetric about the sagittal plane during stable hovering flights; therefore, we focused on the right wing for data acquisition and subsequent analyses. The recorded images were transferred to two Windows laptop computers via Gigabit Ethernet. The experiment was performed under the supervision of the zoo.

A custom-made calibration tool composed of eight steel spheres and connecting rods was used for the three-dimensional camera calibration. The calibration tool had dimensions of 150 mm×120 mm×120 mm (electronic supplementary material, figure S1). The right wing of the hovering hummingbird was confined in this volume during the strokes. The coefficients for stereo reconstruction using direct linear transformation (DLT) were obtained using the commercial software DIPP-Motion Pro version 2.24d (DITECT Corporation, Japan), where the spatial errors were 0.087 mm×0.051 mm×0.10 mm for the local calibration frame coordinates; thus, the RMS error was 0.14 mm. A metallic ruler that was hung in the location of the feeder was separately recorded, providing the vertical vector for horizontal adjustment.

### 2.2. Wing model reconstruction

**Figure 1.**
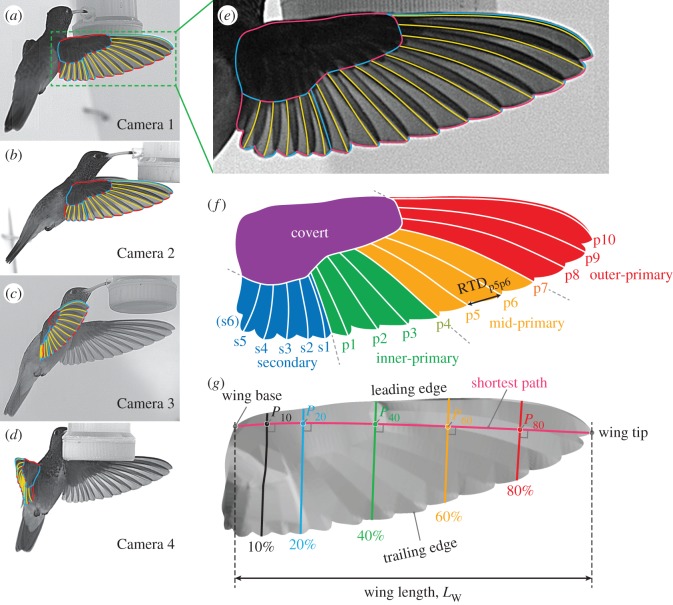
Wing model. (*a*–*d*) Original images with tracked lines at the late downstroke (*t*=0.48*T*). The line segments are indicated by different colours, where yellow lines are the rachides (feather shafts, rachises). Cameras 1, 3 and 4 were Photron SA3 and Camera 2 was Photron SA2. Note the images were cropped to focus on the bird. (*e*) Enlarged view of the Camera 1 image. All these line segments were used for the three-dimensional model reconstruction. (*f*) Illustration of rachides (white lines) and the definitions of the regions. The regions are as follows: purple, Covert region; blue, secondary region; green, inner-primary region; orange, mid-primary region; and red, outer-primary region. The symbols *p*1,*p*2,…,*p*9,*p*10 are the primary feathers and *s*1,*s*2,…,*s*5 are the secondary feathers. The rachis of the 6th secondary (s6) was generally not visible in the recorded images; therefore, it was not tracked. The rachis of the 4th primary (p4) was selected for the boundary between the inner-primary and mid-primary regions. Similarly, the rachis of the 7th primary (p7) was selected for the boundary between the Mid-primary and outer-primary regions. The rachis tip distance (RTD) between the rachis of the 5th primary (p5) and the rachis of the 6th primary (p6) is shown for illustration purposes. (*g*) Reconstructed three-dimensional wing model. The shortest path (magenta) and the four wing cross sections perpendicular to the shortest path are shown. The locations of the wing sections are 10% (black), 20% (blue), 40% (green), 60% (orange) and 80% (red) of the shortest path length *L*_SP_. The intersection of the shortest path and the cross sections are indicated by *P*_10_, *P*_20_, *P*_40_, *P*_60_ and *P*_80_. The wing length *L*_W_ is the distance between the wing base and the wing tip.

After preliminary tracking for obtaining the wingbeat frequency for the four separate flight sequences (bouts) consisting of 112 wingbeat cycles in total, one wingbeat cycle was selected for further study (see electronic supplementary material, figure S2). The selected stroke cycle consisted of 69 image frames. Among these frames, the 2nd, 6th, 10th,…,58th, 62nd and 66th frames (17 frames in total) were selected for wing reconstruction to reduce the tracking time. After applying the unsharp-mask filter, each image was imported to the commercial CAD software Rhinoceros 5 (Robert McNeel & Associates, USA). The wing outline, the boundaries between covert feathers and other parts (hereafter, covert edge), the boundary between primary and secondary feathers, and rachides (feather shafts, yellow lines in figure 1*a*–*e*) were manually traced using the ‘control point curve’ command (electronic supplementary material, figure S3A). Each curve was then extracted as points with equal spacings. The exported curves (points) were then processed using an *in-house* DLT program written in the Fortran 90 programming language, where the coefficients obtained in the three-dimensional calibration were used. Because the identity between camera images for each curve is known only for the start and endpoints, a simplex optimization was used to obtain the three-dimensional curves. The obtained three-dimensional curves were projected back to each of four two-dimensional camera coordinates; then, the deviations of the projected curves and the originally tracked curves were visually examined. The tracked curve was slightly altered and the three-dimensional curves were generated again. This iterative process was repeated until finally obtaining the good match. The electronic supplementary material, video S5, shows the final images where the traced lines are superimposed. The reconstructed curves (points) were then fitted with triangular elements to generate a wing surface model (electronic supplementary material, figure S3B), and through further fitting, a numerical grid with 201×401 grid points (in chordwise and spanwise directions, respectively) was finally obtained (figure 1*g*).

There were minor notches (v-shaped spacings) along the wing trailing edge due to the feather tips. These notches were traced and reproduced as much as possible. However, for a few time frames during early to mid-upstroke, several feathers (around p4 to p1, s1 to s4) were separated from each other in the direction perpendicular to each feather plane (vane). The resulting slits (gaps) were not perfectly traced in the current study for the sake of easier grid generation (see also figure 16). Additionally, note that we do not have information on the wing thickness; thus, the wing model has zero thickness.

### 2.3. Morphological and kinematic parameters

The morphological and kinematic parameters were obtained using the reconstructed wing model to describe and evaluate the wing morphing.

#### 2.3.1. Wing length

Wing length *L*_W_ is defined as the straight line distance between the wing base and wing tip. Note that the wing length *L*_W_ is different from the semi-span (half of the wing span), which is the sum of a single wing length and half of the body width.

#### 2.3.2. Shortest path length

The shortest path is defined as the shortest curve on the wing surface connecting the wing base and wing tip (figure 1*g*, magenta line). The shortest path length *L*_SP_ is always greater than or equal to the wing length *L*_W_ (see electronic supplementary material, figure S4). From an aerodynamics perspective, it may be argued that the wing length can be the better index for a general bird’s (flexing) wings, because the smaller aerodynamic force during upstroke compared to during downstroke due to wing flexing would better be predicted with the instantaneous wing length rather than shortest path length. In fact, we think the *length* of the shortest path may only be useful in calculating the mean chord length (by area/shortest path length), but rather the shortest path itself is very useful in describing other morphing parameters defined below. As we will later show, we chose five wing cross-sectional locations along the shortest path for characteristic points for the wing morphing parameters: 10, 20, 40, 60, 80% *L*_SP_ (plus 100% *L*_SP_, which is the wing tip). The 20, 40, 60 and 80% locations are chosen to show the spanwise variation of the parameters. The 10% location, which is the most proximal location among the selected locations, was chosen because it will be used as the datum for the morphing of the outer sections. The wrist joint probably coincides near the leading edge of the 10% *L*_SP_ section or the intersection of the shortest path and the 10% *L*_SP_ section. This is because the wrist joint is located between one of the arm bones (ulna) and a hand bone, where secondary feathers are attached to the former and primary feathers are attached to the latter. Therefore, extending the boundary between secondary and inner-primary regions towards the covert region would lead to the wrist joint.

#### 2.3.3. Wing area

Wing area *A*_W_ is defined as half of the wing wetted area and calculated from the triangular elements (electronic supplementary material, figure S3B). To assess where the area varies the most in the wing during the stroke cycle, the wing was divided into five regions based on the natural features (figure 1*f*): covert, secondary, inner-primary, mid-primary and outer-primary. The primary regions were selected such that each regional area would have approximately the same area. Note that these regions are based only on the external appearances and do not represent the true anatomy of the wing. The root portions of the flight feathers (primary and secondary feathers) are covered with covert feathers, and we cannot observe them. For example, secondary feathers are in reality considerably longer than how they appear to be in figure 1; therefore, the wing area for the secondary region is largely underestimated compared to the ‘anatomical’ area composed of secondary feathers.

#### 2.3.4. Rachis tip distance

To investigate the cause of the changes in wing area, the distance between the two adjacent rachis tips was calculated for each feather pair and then summed for each region. Hereafter, we will refer to this measure as the rachis tip distance (RTD). The RTD between p5 feather and p6 feather (RTD_*p*5*p*6_) is shown in figure 1*f* as an example. The RTD for the mid-primary region is the sum of RTD_*p*4*p*5_, RTD_*p*5*p*6_ and RTD_*p*6*p*7_. A similar process applies to the other regions.

#### 2.3.5. Single-wing aspect ratio and mean chord length

The single-wing aspect ratio *AR*_s_ indicates how slender a wing is on average, and it is defined as ${\mathrm{AR}}_{s}:={L_{\mathrm{SP}}}^{2}/A_{W}$. The mean chord length *c*_m_ is defined as *A*_W_/*L*_SP_; therefore, the aspect ratio can also be written as *AR*_s_=*L*_SP_/*c*_m_.

#### 2.3.6. Stroke plane angle, body angle and anatomical stroke plane angle

**Figure 2.**
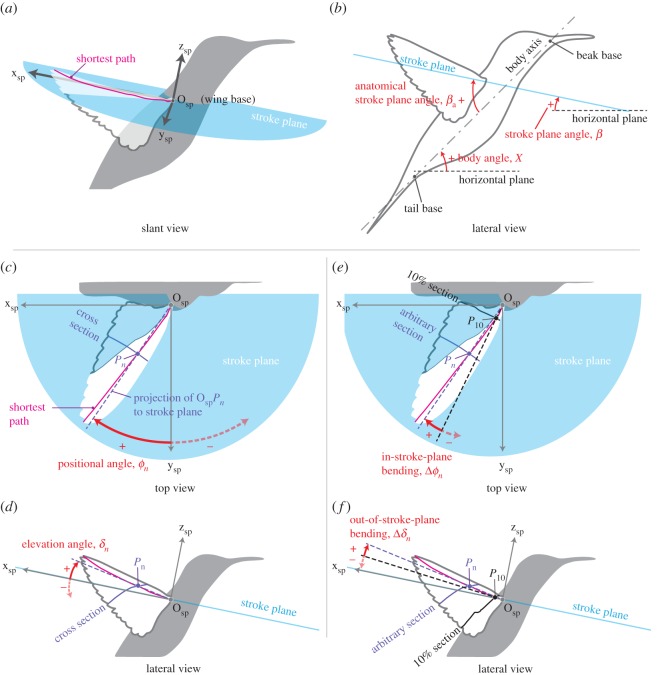
Schematics illustrating the stroke plane related angles. In (*a*), the slant view of the bird and the stroke plane coordinates *O*_sp_-*x*_sp_*y*_sp_*z*_sp_ are shown. In (*b*), the stroke plane angle *β*, body angle *χ* and anatomical stroke plane angle *β*_a_ are shown. In (*c*) and (*d*), the positional and elevation angles, respectively, are shown. In (*e*) and (*f*), the in-stroke-plane bending and out-of-stroke-plane bending, respectively, are shown.

The stroke plane is defined by the linear-regression path for the wing tip in the lateral view of the bird (for the determination of the global coordinates, see the electronic supplementary method). The stroke plane angle *β* is the angle between the stroke plane and the horizontal plane. Choosing a different point from the wing tip changed the stroke plane angle by only 2^°^ (see electronic supplementary material, figure S5). As we are focusing on only the right wing, we chose the right wing base as the origin *O*_sp_ for the stroke plane coordinates (frame). The *z*_sp_-axis is perpendicular to the stroke plane; the *y*_sp_-axis is on the stroke plane and perpendicular to the sagittal plane, and it points to the right of the bird; and the *x*_sp_-axis is on the stroke plane and parallel to the sagittal plane, and it points to the dorsal side of the bird (figure 2*a*). As this study focuses on the wing kinematics and morphing, the body angle *χ*, which is the angle between the horizontal plane and body axis, was only roughly measured, and the body axis was approximated by the line connecting the base of the beak and the base of the tail (rectrices; figure 2*b*). The anatomical stroke plane angle *β*_a_ is the angle between the body axis and the stroke plane (figure 2*b*).

#### 2.3.7. Positional angle and elevation angle

The positional angle *ϕ* and elevation angle *δ* describe the in-stroke-plane and out-of-stroke-plane kinematics, respectively, of a characteristic point *P* on the shortest path. The positional angle is the angle between the *y*_sp_-axis and the projection of the line *O*_sp_*P* to the stroke plane, and *ϕ*>0 when *P* is on the dorsal side of the body, whereas *ϕ*<0 when *P* is on the ventral side (figure 2*c*). The elevation angle is the angle between the stroke plane and the line *O*_sp_*P*, and *δ*>0 when *P* is above the stroke plane, whereas *δ*<0 when *P* is below the stroke plane (figure 2*d*). We chose the 10, 20, 40, 60, 80 and 100% *L*_SP_ locations for *P*, where 100% *L*_SP_ is the wing tip. We hereafter refer to the positional and elevation angles at the *n*% *L*_SP_ location as *ϕ*_*n*_ and *δ*_*n*_, respectively.

#### 2.3.8. Wingbeat amplitude, wingbeat period and wingbeat frequency

The wingbeat amplitude *Φ* is the absolute difference between the maximum and minimum positional angles during the stroke cycle. The wingbeat period *T* was calculated by fitting a sinusoidal curve to the measured time history of positional angle with the Levenberg–Marquardt algorithm. The maximum positional angle did not coincide with any image frame; thus, the tracked frames (every 4th frame from the 2nd to the 66th frame) correspond to 0.019*T*, 0.077*T*, 0.13*T*, …, 0.82*T*, 0.88*T* and 0.94*T* with a uniform time interval of 0.0575*T*. The wingbeat frequency *f* is the inverse of the wingbeat period *T*, i.e. *f*=1/*T*.

#### 2.3.9. Spanwise bending

The spanwise bending is defined as the difference in the positional or elevation angle at an arbitrary spanwise location *P*_*n*_ against the angle for the 10% shortest path length location *P*_10_ (figure 2*e*,*f*). The spanwise bending has two components: in-stroke-plane Δ*ϕ* (figure 2*e*) and out-of-stroke-plane Δ*δ* (figure 2*f*). These components are defined as follows: Δ*ϕ*_*n*_:=*ϕ*_*n*_−*ϕ*_10_ and Δ*δ*_*n*_:=*δ*_*n*_−*δ*_10_, where *n*=20, 40, 60, 80, 100 are the shortest path locations as in the definitions of the positional and elevation angles.

#### 2.3.10. Local bending gradient

The local bending gradients Δ*ϕ*/Δ*L*_SP_ and Δ*δ*/Δ*L*_SP_ for the in-stroke-plane direction and out-of-stroke-plane direction, respectively, were calculated to determine the most prominent locations of the bending. They are defined as the spatial gradients of the positional angle and elevation angle along the spanwise (shortest-path wise) direction and calculated using the second-order central difference scheme; therefore, note that they both have the dimension of angle/length (cf. the spanwise bending has the dimension of angle). For the in-stroke-plane direction, Δ*ϕ*_*n*_/Δ*L*_SP_:=(*ϕ*_*n*+10_−*ϕ*_*n*−10_)/(0.2*L*_SP_), where *n* represents the per cent shortest path length and similarly for the out-of-stroke-plane direction. For example, the local in-stroke-plane bending gradient at the 20% *L*_SP_ location is obtained by Δ*ϕ*_20_/Δ*L*_SP_:=(*ϕ*_30_−*ϕ*_10_)/(0.2*L*_SP_). Note that theoretically, an infinitesimally small distance (width) along the spanwise direction should be used, but it would be too prone to the noise; therefore, we chose the distance of 20% *L*_SP_.

#### 2.3.11. Wing cross section, chord length and camber

**Figure 3.**
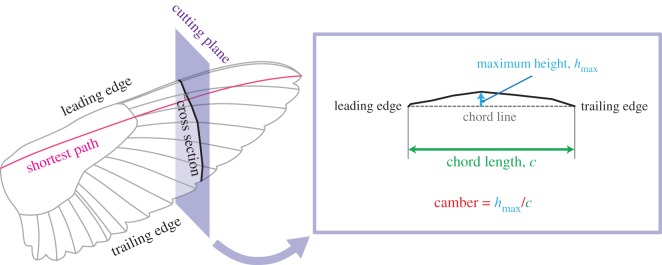
Wing cross section, chord length and camber.

The wing cross section (wing section, also known as aerofoil or airfoil) was obtained by slicing the three-dimensional wing model with a cutting plane perpendicular to the shortest path at an arbitrary location (figure 3). The locations at the 10, 20, 40, 60 and 80% shortest path lengths were selected. The chord length *c* is the distance between the leading edge and trailing edge for each wing cross section (figure 3). Camber may be defined as the height (distance) from the chord line to the wing cross section. To compare the cambers at different spanwise locations or at different time frames, we normalized the height *h* with the chord length *c* for each cross section (i.e. *h*/*c*). Moreover, one needs to choose the height at a certain chordwise location to represent each cross section. Within the animal flight literature, it appears that there are mainly two options for this height: the maximum height [17,37] or the height at mid-chord [38,39]. In this study, we chose the maximum height, *h*_max_. Hereafter, the maximum camber normalized by the chord length, $h_{\max}/c$, will be referred to as camber (figure 3). See electronic supplementary material, figure S6 and table S1 for the cambers at mid-chord.

#### 2.3.12. Angle of incidence

**Figure 4.**
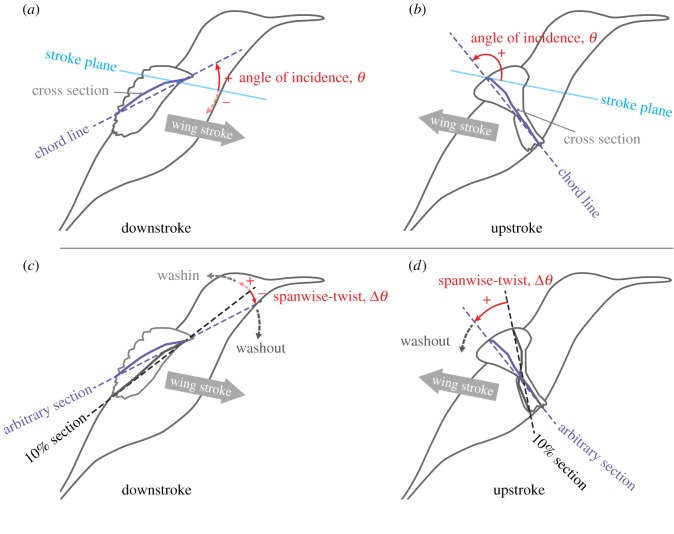
Definitions of angle of incidence (*a*,*b*) and spanwise twist (*c*,*d*). In (*c*,*d*), the spanwise twist for the 40% *L*_SP_ section are shown as examples. The spanwise twist for the other locations were similarly defined and calculated. In the present hovering, generally the AoI was less than 90^°^ during downstroke (*a*) and more than 90^°^ during upstroke (*b*). Also, the spanwise twist was negative during downstroke (*c*) and positive during upstroke (*d*).

Angle of incidence (AoI) *θ* is defined as the angle between a chord line and the stroke plane (figure 4*a*,*b*). The leading edge of a wing cross section is pointing forwards (and generally above the stroke plane) when 0^°^≤*θ*≤90^°^, whereas it is pointing backwards (and typically above the stroke plane) when 90^°^≤*θ*≤180^°^. Note that the angle of incidence is occasionally referred to as the geometric AoA. We avoided using this name because we believe that it is confusing with the kinematic AoA or effective AoA explained later. We will refer to the change in the AoI for the entire wing as torsional rotation and the changes in the signs of AoI at stroke reversals as torsional reversals.

#### 2.3.13. Spanwise twist

The spanwise twist Δ*θ* is defined as the difference in the AoIs at a spanwise location and at the 10% *L*_SP_ location, i.e. Δ*θ*_*n*_:=*θ*_*n*_−*θ*_10_, where *n*=20,40,60,80 are the per cent shortest path locations (figure 4*c*,*d*; (*c*) is during downstroke and (*d*) is during upstroke).

#### 2.3.14. Local twist gradient

The local twist gradient Δ*θ*/Δ*L*_SP_ is the spatial gradient of the AoI along the spanwise (shortest-path wise) direction, which was calculated similarly to the local bending gradient, i.e. Δ*θ*_*n*_/Δ*L*_SP_:=(*θ*_*n*+10_−*θ*_*n*−10_)/(0.2*L*_SP_), where *n* represents the per cent shortest path length. For example, the local twist gradient at the 20% *L*_SP_ location is obtained by Δ*θ*_20_/Δ*L*_SP_:=(*θ*_30_−*θ*_10_)/(0.2*L*_SP_). Note this has the dimension of angle/length as the local bending gradient does.

#### 2.3.15. Kinematic angle of attack

**Figure 5.**
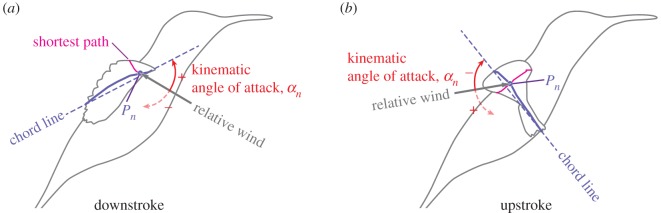
Kinematic angle of attack (AoA). The point *P*_*n*_ is the intersection of the shortest path and the wing cross section at *n*% *L*_SP_, as in the figure 2. Generally, the AoA was positive during downstroke (*a*) and negative during upstroke (*b*).

Kinematic angle of attack (AoA) *α* is defined as the angle between a chord line and the relative wind vector against the wing section. For a flapping wing, this definition is still ambiguous because the relative wind is not uniform for the wing section, and one needs to choose a specific point to obtain the relative wind vector. We chose *P*_*n*_ for this point, which is the intersection between a wing cross section and the shortest path at the *n*% *L*_SP_, which is exactly the same point used for obtaining the positional and elevation angles (figure 5; see electronic supplementary material, figure S7 for a more detailed schematic). In practice, we first obtained the velocity vector for the *P*_*n*_ using the second-order central difference scheme in time. For simplicity, we used the raw time frames rather than interpolating the coordinates over time, which would generally be preferred before taking a temporal derivative. Therefore, we present the AoA for 15 time frames (from 0.077*T* to 0.88*T*). The relative wind vector was then obtained by simply changing the sign of the velocity vector for the *P*_*n*_ (note that if there is any uniform wind, one needs to consider the vector summation). Note that a positive AoA means that the relative wind is coming from the ventral side of the wing, whereas a negative AoA means that the relative wind is coming from the dorsal side of the wing. Moreover, note that we appended the word *kinematic* to clarify that our definition is different from either the angle of incidence (which is occasionally called the geometrical AoA) or the effective AoA, which incorporates the effect of induced velocity.

## 3. Results

### 3.1. Wing model

**Figure 6.**
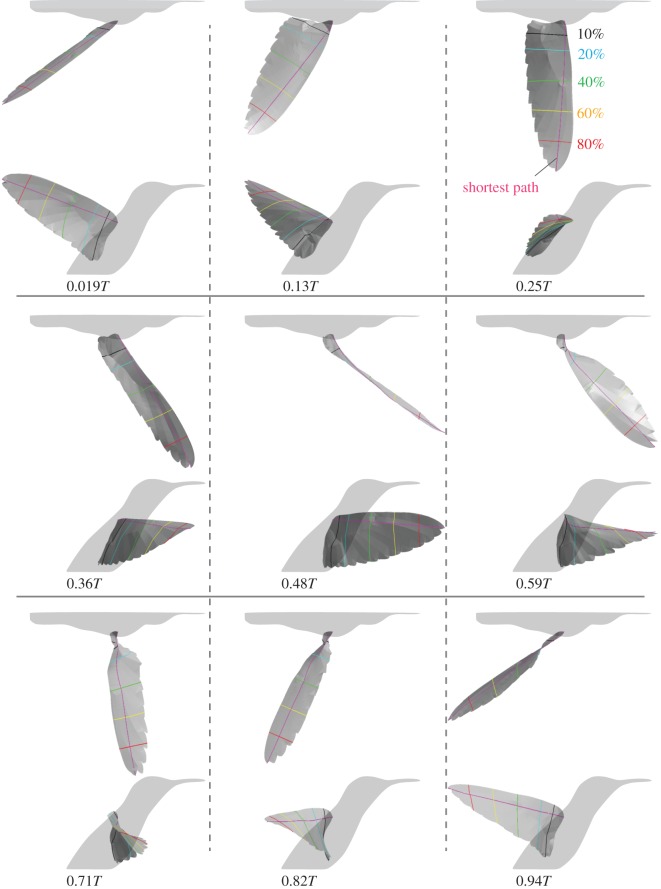
Top and lateral views of the reconstructed wing model. Nine time frames among 17 tracked frames are shown. See electronic supplementary material, video S6 for the complete cycle.

Nine selected time frames (among 17 frames) for the reconstructed wing model are shown in figure 6, where the wingbeat period is *T*=0.0348 s (see electronic supplementary material, video S6 for the complete cycle). The dynamic wing morphing is visually observable. For instance, substantial twist is apparent during upstroke (figure 6, *t*=0.59*T*, 0.71*T* and 0.82*T*). Note that time *t*=0*T* is set as the beginning of downstroke, 0.5*T* coincides with the end of downstroke (and beginning of upstroke) and 1.0*T* is the end of downstroke (and beginning of the next downstroke). Hereafter, we call the time period around 0*T* and 1.0*T* the pronation phase or simply pronation, and we call that around 0.5*T* the supination phase or simply supination.

The basic wing morphological and kinematic parameters extracted from the wing model are presented in table 1.

**Table 1.** Basic morphological and kinematic parameters. The symbols with overbars (wing length, shortest path length, wing surface area, single-wing aspect ratio and mean chord length) are the averaged values over one wingbeat period *T*.

wingbeat frequency	*f*	28.8 (Hz)
wingbeat amplitude	*Φ*	103 (^°^)
stroke plane angle	*β*	11.7 (^°^)
wing length	${\bar{L}}_{W}$	69.3 (mm)
shortest path length	${\bar{L}}_{\mathrm{SP}}$	70.0 (mm)
wing surface area	${\bar{A}}_{W}$	1365 (mm^2^)
single-wing aspect ratio	${\bar{\mathrm{AR}}}_{s}$	3.53
mean chord length	${\bar{c}}_{m}$	19.5 (mm)

### 3.2. Lengths and aspect ratio

**Figure 7.**
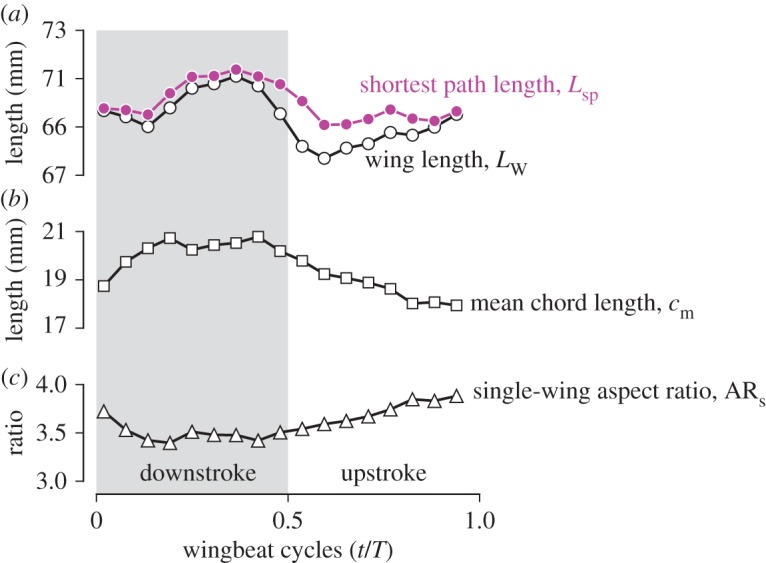
Instantaneous values of (*a*) shortest path length (magenta filled circles) and wing length (black open circles), (*b*) mean chord length (black open squares) and (*c*) single-wing aspect ratio (black open triangles). Grey shaded region indicates downstroke period.

The time courses of the shortest path length *L*_SP_ and wing length *L*_W_ are shown in figure 7*a* (magenta filled circles and black open circles, respectively). Both lengths take their maximum values at late downstroke and their minimum values at early upstroke, but the peak-to-peak variations are less than 4 mm, while the wingbeat cycle average lengths were approximately 70 mm (table 1), i.e. the variations were less than 5%. The ratio of the wing length at mid-upstroke (0.77*T*) to the wing length at mid-downstroke (0.25*T*) was 0.97. This result is close to the span ratio of 98±4% for the hovering rufous hummingbirds [4], although the definitions are slightly different. The span ratio includes the body width and is affected by the positional angle and the elevation angle at mid-strokes if they are non-zero.

**Figure 8.**
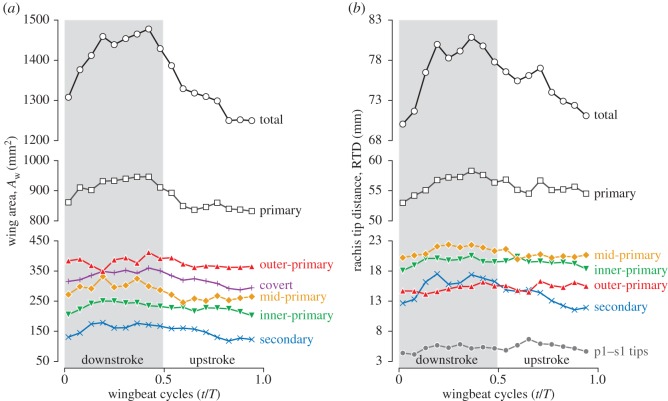
Instantaneous values of wing surface area (*a*) and RTD (*b*). Black open circles, total (entire wing); black open squares, primary region; purple plus symbols, covert region (only in *a*); blue crosses, secondary region; red triangles, outer-primary region; orange diamonds, inner-primary region; green inverted triangles, mid-primary region; and grey filled circles, distance between p1 tip and s1 tip (only in *b*).

The mean chord length *c*_m_ substantially varied throughout the stroke (black open squares in figure 7*b*). In general, the mean chord length increased during downstroke and decreased during upstroke (maximum of 20.8 mm at 0.42*T* and minimum of 17.9 mm at 0.94*T*), and the peak-to-peak ratio was 1.16. This trend is quite similar to the trend of the wing area (black open circles in figure 8*a*), which is natural because the variation in the shortest path was small. This result is also reflected in the single-wing aspect ratio *AR*_s_ (figure 7*c*), where *AR*_s_ decreased during downstroke and increased during upstroke (minimum 3.40 at 0.19*T* and maximum 3.88 at 0.94*T*).

### 3.3. Wing area and rachis tip distance

The temporal variation in wing area is shown in figure 8*a*. The total wing area (black open circles) *A*_*w*_ exhibited substantial variation within a stroke cycle. At the beginning of downstroke (at 0.019*T*), the area was 1307 mm^2^, and it rapidly increased during the early downstroke to 1459 mm^2^ at 0.19*T*. The area was generally kept constant and peaked at 0.42*T* with 1478 mm^2^. During supination to early upstroke, the area rapidly decreased to 1329 mm^2^, which is already close to the value at the beginning of downstroke. During mid-upstroke to late upstroke, the area gradually decreased and the minimum area of 1250 mm^2^ was observed at the end of upstroke (0.94*T*).

 Table 2 shows the mean (wingbeat-cycle averaged), maximum (at 0.42*T*) and minimum (at 0.94*T*) wing areas and the ratios at these time frames. The values in the parentheses in the ‘mean’ column represent the fraction of the total wing area in percentages. Note that 0.42*T* and 0.94*T* are not necessarily the maximum and minimum timings for each wing region. The maximum and minimum areas for each region, along with their ratio, are shown in table 3. The primary region contributed to approximately two-thirds of the total wing area, whereas the covert region contributed one-fourth and the secondary region contributed only one-tenth of the total wing area. The peak-to-peak variation for the total wing area was 18% (table 2). Interestingly, the variation in the primary region was 14%, whereas the variations in the secondary and covert regions were 53% and 22%, respectively (table 3).

**Table 2.** Time-average wing areas, peak wing areas and the ratios between the peaks, along with the contribution from each wing region. The per cent values in the parentheses in the ‘mean wing area’ column are the fraction of each region against the total wing area at each time instance.

	wing area (mm^2^)			ratio
	__________________________________			_________________
region	mean	at 0.42*T*	at 0.94*T*	(*A*_*w*,0.42*T*_/*A*_*w*,0.94*T*_)
total	1365	1478	1250	1.18
covert	326 (24%)	360	294	1.22
secondary	152 (11%)	171	123	1.40
primary	887 (65%)	946	832	1.14
inner	229 (17%)	235	202	1.16
mid	281 (21%)	300	265	1.13
outer	377 (28%)	412	365	1.13

**Table 3.** The maximum and minimum wing areas for each region.

	wing area (mm^2^)			ratio
	_______________________________________			____
region	mean	maximum	minimum	$A_{w,\max}/A_{w,\min}$
total	1365	1478 (0.42*T*)	1250 (0.42*T*)	1.18
covert	326	360 (0.42*T*)	287 (0.88*T*)	1.25
secondary	152	179 (0.19*T*)	117 (0.83*T*)	1.53
primary	887	946 (0.42*T*)	832 (0.94*T*)	1.14
inner	229	250 (0.25*T*)	202 (0.94*T*)	1.24
mid	281	332 (0.19*T*)	246 (0.59*T*)	1.35
outer	377	412 (0.42*T*)	349 (0.19*T*)	1.18

The overall trends in the RTD (figure 8*b*) are similar to the trends in the wing area, i.e. rapid increase at early downstroke, beginning to decrease at late downstroke and continue to decrease throughout upstroke.

### 3.4. Wing tip path, stroke plane angle and anatomical stroke plane angle

**Figure 9.**
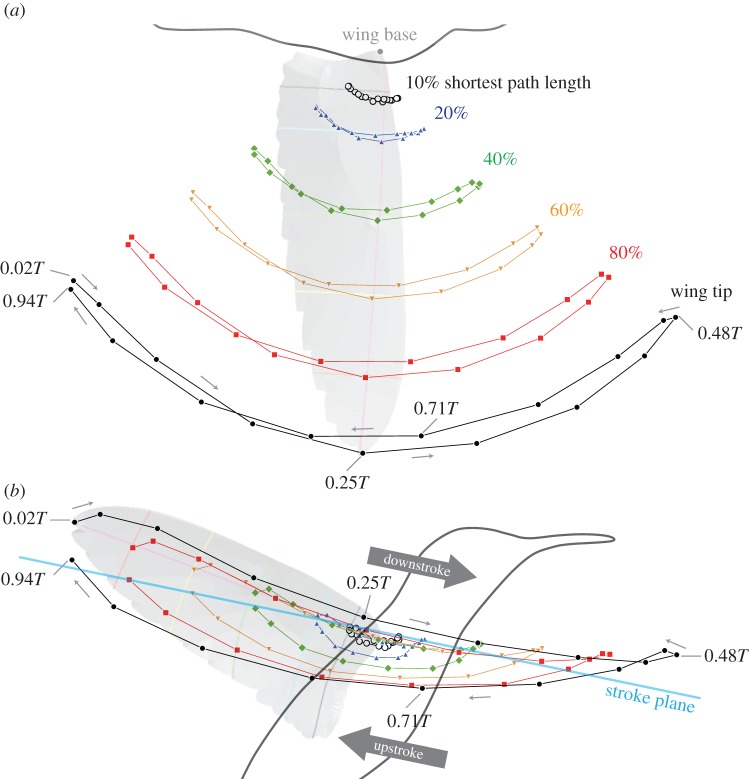
Top (*a*) and lateral (*b*) views for the paths of six characteristic points along the shortest path (*P*_*n*_). Black open circles, 10% *L*_SP_ (shortest path length); blue triangles, 20% *L*_SP_; green diamonds, 40% *L*_SP_; orange inverted triangles, 60% *L*_SP_; red squares, 80% *L*_SP_; black filled circles, wing tip. In (*b*), horizontal plane (black line), stroke plane (blue line) and stroke plane angle (red) are illustrated.

The paths for the points *P*_*n*_, the intersection of the shortest path and the wing cross section, where *n*=10, 20, 40, 60, 80% *L*_SP_ (figure 2), are shown in figure 9. The blue line in figure 9*b* is the stroke plane, and the stroke plane angle *β* was 11.7^°^.

The body angle *χ* was approximately 35^°^ at the beginning of drinking for the selected flight sequence, and it gradually increased until approximately 45^°^ at the wingbeat period that we selected. Consequently, the anatomical stroke plane angle *β*_a_ during the selected wingbeat cycle was approximately 57^°^. These angles are slightly smaller than the angles found for the hovering rufous hummingbirds [4]. We also found that the body angle apparently oscillated approximately 1–2^°^ for each half stroke and that the oscillation was larger during downstroke than that during upstroke, indicating aerodynamic force asymmetry.

### 3.5. Positional angle, elevation angle and spanwise bending

**Figure 10.**
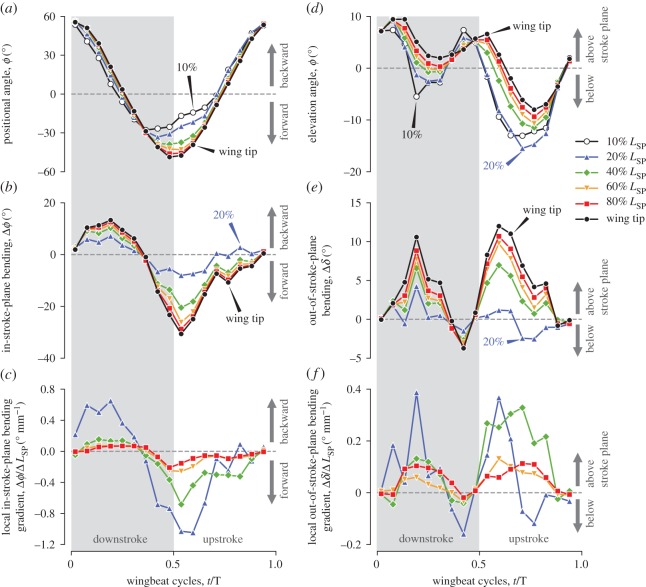
Positional angle (*a*), in-stroke-plane spanwise bending (*b*), local in-stroke-plane bending gradient (*c*), elevation angle (*d*), out-of-stroke-plane spanwise bending (*e*) and local out-of-stroke-plane bending gradient (*f*). Grey shaded regions indicate the downstroke period. Black open circles, 10% *L*_SP_ section; blue triangles, 20% *L*_SP_ section; green diamonds, 40% *L*_SP_ section; orange inverted triangles, 60% *L*_SP_ section; red squares, 80% *L*_SP_ section; and black filled circles, wing tip.

The positional angle for the wing tip shows a sinusoidal shape (figure 10*a*, black filled circles). The durations of downstroke and upstroke were almost identical. The dorsal and ventral amplitudes were both approximately 50^°^. The sinusoidal trend can also be observed for the distal wing sections (figure 10*a*, red squares and orange inverted triangles). Interestingly, however, the positional angles for the proximal wing sections are non-sinusoidal during supination (figure 10*a*, blue triangles and black open circles). A phase difference was observed along the spanwise locations during downstroke and during supination, where the proximal sections are always advanced relative to the distal sections. Specifically, the positional angle for the 10% *L*_SP_ takes its minimum (most forward position) before supination (at approximately 0.36*T*–0.42*T*), but the wing tip reached its minimum at 0.5*T* by definition; therefore, there is approximately a 0.1*T* delay for the wing tip compared to the wing base. During upstroke, the proximal sections advanced again, but at the pronation, there was no phase difference.

The elevation angle for the wing tip (figure 10*d*, black filled circles) also has a sinusoidal shape, but its frequency is twice that of the positional angle. The phase of the wing tip elevation angle is also slightly delayed compared with the positional angle, i.e. the upward peaks are observed shortly after pronation and supination, whereas the downward peaks are observed shortly after the mid-strokes. The phase delay along the spanwise locations is also apparent, and the proximal sections are advanced compared to the distal sections.

The in-stroke-plane bending and the out-of-stroke-plane bending are shown in figure 10*b* and 10*e*, respectively. Note that, in both plots, the values for the 10% *L*_SP_ are not shown because it is always 0^°^ by definition. There was no bending at pronation (0.019*T*) or at supination for the out-of-stroke-plane direction (0.48*T*, figure 10*e*). However, at the other time instances the distal portion of the wing was delayed compared to the proximal portion; hence, the wing was bent. The in-stroke-plane bending has a positive (backward bending) peak at mid-downstroke (for the wing tip, 13.3^°^ at 0.19*T*) and a negative (forward bending) peak at early upstroke (for the wing tip, −30.6^°^ at 0.54*T*). The out-of-stroke-plane bending has two positive (above stroke plane bending) peaks of approximately 10^°^ at mid-downstroke and early upstroke. There was nearly no negative (below stroke plane bending) peak. Therefore, in contrast to the in-plane bending where both forward and backward bending were observed, the out-of-stroke-plane bending occurred mostly towards the upward direction only. We will discuss the potential cause of these results in the Discussion section.

The local bending gradients were generally larger in the proximal portion of the wing than in the distal portion for both in- and out-of-stroke-plane directions (figure 10*c*,*f*). These gradients showed asymmetry in two half-strokes. During downstroke, the most proximal location (20% *L*_SP_, blue triangles in figure 10*c*,*f*) contributed the most to the bending, which is particularly clear for the in-stroke-plane direction (figure 10*c*). However, during upstroke, the 40% location (green diamonds in figure 10*c*,*f*) also contributed to the bending. In both half strokes, the contributions from the distal (60 and 80% *L*_SP_) locations were minor.

### 3.6. Wing cross section

**Figure 11.**
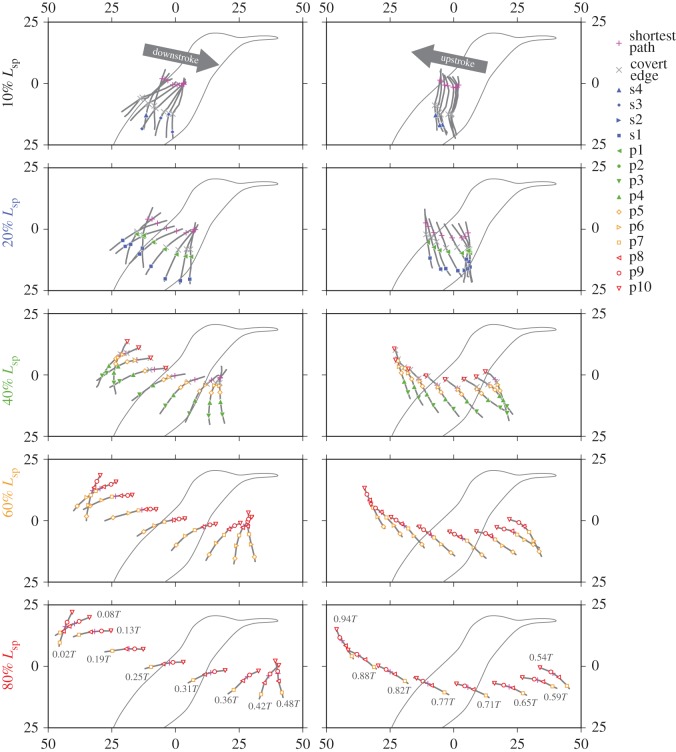
Instantaneous wing sections at five spanwise locations through a wingbeat cycle (left column, downstroke; right column, upstroke), views from the right side of the bird. The position of the shortest path (magenta cross symbol) for each wing section is accurate in the *xz*-plane, where the numbers in both axes are distances from the wing base in millimetres (the wing base is fixed in space for all the time frames). Each wing cross-sectional shape including symbols is the same as the shape in figure 12, but the section in this figure is rotated around the shortest path, such that the cross-sectional plane is aligned to the global *xz*-plane (sagittal plane).

**Figure 12.**
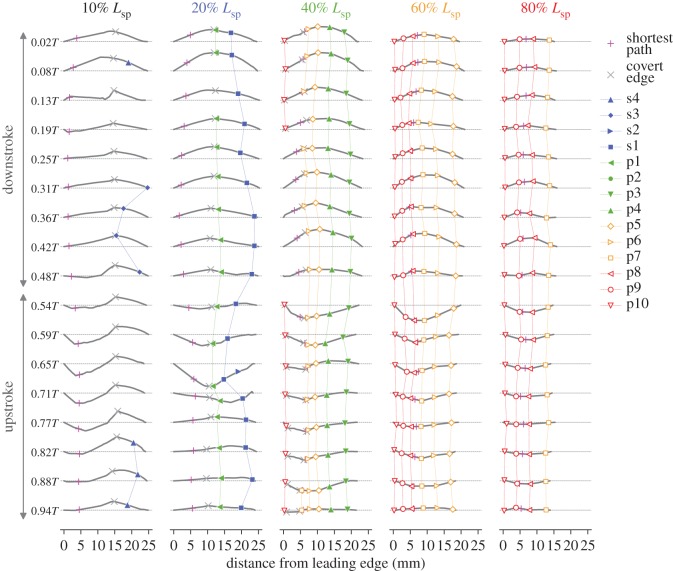
Time series of wing cross sections at five spanwise locations (10% *L*_SP_ in the leftmost column to 80% *L*_SP_ in the rightmost column). Each section is placed such that the chord line is horizontally aligned and the leading edge points to the left; therefore, the readers are viewing the wing from the wing base. The horizontal and vertical scales are the same, i.e. not enlarged vertically.

The wing cross sections at five spanwise locations are presented in figures 11 and 12. In figure 11, the bird is viewed from the lateral right side. The magenta symbols are the intersection between the cross section and the shortest path. The other coloured symbols indicate the intersection between the cross section and the characteristic lines such as rachides. The grey cross symbols represent the boundary between the covert region and the other regions (covert edge). The wing outlines (leading edge or trailing edge) are not marked by the symbols. Note the shortest path (magenta cross symbols) is not an anatomical feature, but the others are anatomical features.

Generally, the 10 and 20% *L*_SP_ wing cross sections contained less rachides because, at proximal sections, the rachides were nearly parallel to the cross sections, whereas at distal sections, the rachides were oblique or even nearly perpendicular to the cross sections (figure 1*f*,*g*). More than half of the 10% *L*_SP_ section and nearly half of the 20% *L*_SP_ section were composed of covert feathers. The kinks near the covert edge (grey cross symbols) in the 10% *L*_SP_ section are probably artefacts due to the reconstruction (no information inside of the covert region) and should not be considered as the true shapes. The 20% *L*_SP_ section contained the s1 rachis (blue filled squares) for all the time instances and p1 rachis (green left-facing filled triangles) for all time instances except 0.13*T*. The s2 rachis (blue right-facing filled triangle) appeared only in the single time frame during upstroke (0.65*T*). The tiny kink that appeared near the trailing edge at 0.71*T* should be an artefact. The 40% *L*_SP_ section contained the p3, p4 and p5 rachides (green bottom-facing filled triangles, green top-facing filled triangles, and orange open diamonds, respectively) for all the time instances and the p6 rachis (orange right-facing open triangles) for all but one time instance (0.19*T*). The 60% *L*_SP_ section contained the p5 to p10 rachides for all time instances, and the 80% *L*_SP_ section contained the p7 to p10 rachides for all time instances. These sections did not cross the covert region and were composed only of primary feathers, indicating that no bony structures were involved (note that the reverse is not necessarily true, i.e. containing covert region does not necessarily mean that the bones are included because the roots of the flight feathers are covered with the covert feathers).

Apparently, on each cross section, the rachides were not always uniformly distributed. Rather, the rachides were weakly clustered near the mid-chord (just after the covert edge) for the 40% *L*_SP_ section, whereas for the 60% *L*_SP_ section, they were clustered between the leading edge and the mid-chord and for the 80% *L*_SP_ section, they were not clustered but uniformly distributed. These variations in the chordwise rachis distribution may be related to the spanwise and chordwise aerodynamic force distributions. Near the leading edge, the strong aerodynamic force due to the LEV would be available during mid-downstroke and mid-upstroke; thus, structural supports from bone/muscle/tendon (for proximal wing sections) or clustered rachides (distal wing sections) would be beneficial for withstanding such forces.

### 3.7. Camber

**Figure 13.**
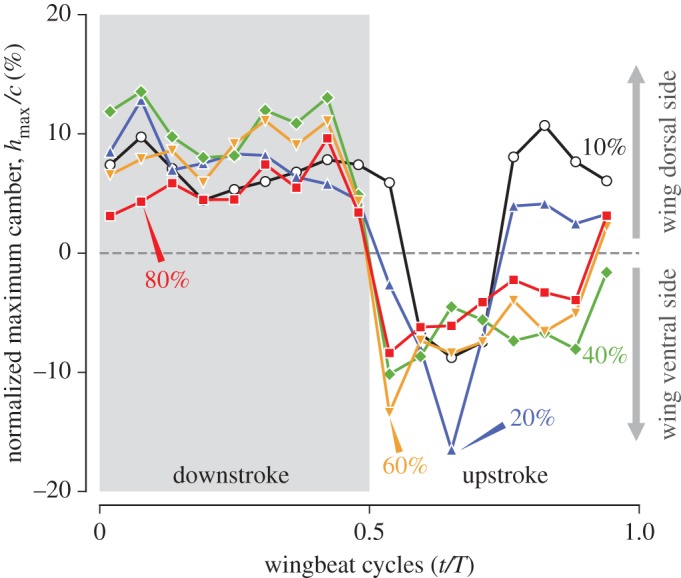
Instantaneous cambers (normalized maximum height, *h*_max_/*c*) at five wing sections. Grey shaded region indicates downstroke period.

One of the most prominent features in the wing cross sections is the temporal variation in the cambers: during downstroke, the wing sections were convex towards the dorsal/backward side, whereas during upstroke, they were convex towards the ventral/forward side (figures 11–13). This feature is also apparent in table 4, which summarizes the half-stroke average camber for wing sections, where all the sections were positive during downstroke, while all but the most proximal section were negative during upstroke. During stroke reversals, cambers sharply changed their signs almost simultaneously for all the wing sections.

**Table 4.** Cambers at five wing sections, averaged over downstroke period and over upstroke period.

section	${\left(\bar{h_{\max}/c}\right)}_{\mathrm{down}}$ (%)	${\left(\bar{h_{\max}/c}\right)}_{\mathrm{up}}$%
10% *L*_SP_	6.9	1.9
20% *L*_SP_	7.7	−2.5
40% *L*_SP_	10.2	−6.6
60% *L*_SP_	8.2	−6.3
80% *L*_SP_	5.4	−3.9

For proximal sections, the maximum camber (inspecting along the chordwise direction) occurred near the covert edge (grey cross symbols in figures 11 and 12). This result is particularly clear for the 20% *L*_SP_ section. The strong negative (ventral) camber during early- to mid-upstroke at *t*=0.65*T* (figure 13, blue triangle) can be correlated with this chordwise location, because at this time instance, the leading edge already started to sweep back but the secondary feathers did not; therefore, a strong camber emerged with the covert edge being the apex. Note, however, that we have no geometrical information within the covert region, and it is possible that the maximum camber is in reality located closer to the leading edge. For the 40% *L*_SP_ section, the covert edge was not the apex (maximum camber) for most of the time instances. Rather, the 5th primary (p5) typically constituted the maximum camber. For the distal sections (60% and 80% *L*_SP_), the covert edge was not included at all, and either p7, p8 or p9 constituted the maximum camber.

### 3.8. Twist

#### 3.8.1. Angle of incidence

**Figure 14.**
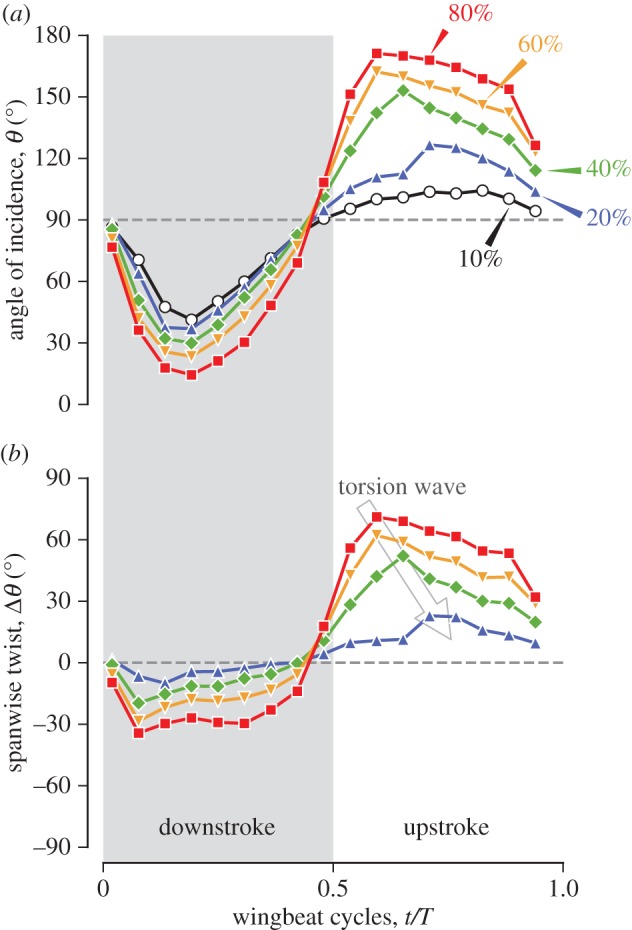
Instantaneous AoIs *θ* (*a*) and spanwise twists (*b*) at wing sections. In (*a*), the dashed line at 90^°^ indicates the perpendicular upward direction to the stroke plane. In (*b*), the arrow indicates the torsion wave propagation. Grey shaded region indicates the downstroke period.

During downstroke (excluding pronation and supination), the angles of incidence (AoIs) for wing sections were kept between 0^°^ and 90^°^, and the angles for more distal sections were always smaller than those for the more proximal sections (figure 14*a*). As will be revisited later, this type of twist with a monotonically decreasing AoI distribution along the wing long axis is called washout. At the beginning of downstroke (0.019*T*), the wing had almost no twist (i.e. nearly flat), and the wing plane was nearly perpendicular to the stroke plane with the leading edge pointing upwards (87.8^°^ at 20% *L*_SP_ and 76.7^°^ at 80% *L*_SP_). The angles decreased in the early downstroke, and local minima were reached at mid-downstroke (0.19*T*) for all the wing sections. During the second half of the downstroke, the angles increased again. The torsional reversal occurred instantly for all the wing sections at the end of downstroke between 0.42*T* and 0.48*T*, i.e. slightly before stroke reversal. In contrast to downstroke, where the temporal waveforms for the AoIs were sinusoidal, the waveforms for the distal AoIs during upstroke were more quadrilateral, i.e. quickly reaching the high angles at early upstroke and gradually decreasing until late upstroke. This result is somewhat similar to the AoIs for insect wings (e.g. orange dashed lines in fig. 3 of [10], where the definition of the feathering angle is different by 90^°^ from the AoI in the current study). The trends were similar for all the wing sections, but the wing proximal sections were delayed to the distal sections, i.e. the maximum AoIs occurred at 0.59*T* for 80% *L*_SP_ (171^°^) and 60% *L*_SP_ (162^°^), at 0.65*T* for 40% *L*_SP_ (153^°^) and at 0.71*T* for 20% *L*_SP_ (127^°^). In contrast to downstroke, the most proximal wing section (10% *L*_SP_ section) did not considerably vary and maintained a nearly constant AoI of approximately 100^°^ (maximum 104^°^ at 0.82*T*).

#### 3.8.2. Spanwise twist

The spanwise twist Δ*θ*_G_ was negative during downstroke and positive during upstroke (figure 14*b*). During downstroke, the twist had monotonically decreasing trends from the proximal to distal wing sections, i.e. washout. Consequently, the leading edge of a certain section was pointing more downwards compared to a more proximal section. All the sections crossed zero spanwise twist between 0.42*T* and 0.48*T* (torsional reversal). During upstroke, the sign was reversed, and the distal section had a larger spanwise twist. The wing was flipped upside down due to the torsional reversal (the wing ventral side was facing upwards); therefore, the leading edge of a distal wing section was pointing more downwards than the leading edge of a more proximal wing section, which is again washout. Generally, washout is preferred for artificial wing-like objects such as propeller blades to suppress the stall near the wing tip by lowering the AoA. It can also be observed that the twist amplitude was larger in the upstroke than in the downstroke. Specifically, the 80% *L*_SP_ section maintained approximately −30^°^ during downstroke, whereas the section reached more than 60^°^ at early upstroke, and then it began gradually decreasing but decreased to 30^°^ only at the end of upstroke.

#### 3.8.3. Local twist gradient

**Figure 15.**
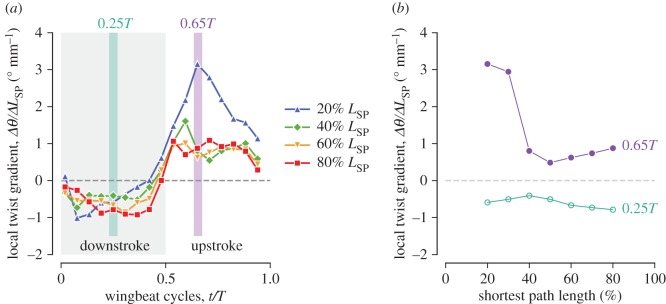
Local twist gradients. In (*a*), the instantaneous values for the four wing sections, where the vertical colour bands correspond to the two representative time frames at mid-downstroke (0.25*T*) and early upstroke (0.65*T*). In (*b*), the spanwise variations of the local twist gradient for these time frames are shown, where green open circles are 0.25*T* and purple filled circles are 0.65*T*.

**Figure 16.**
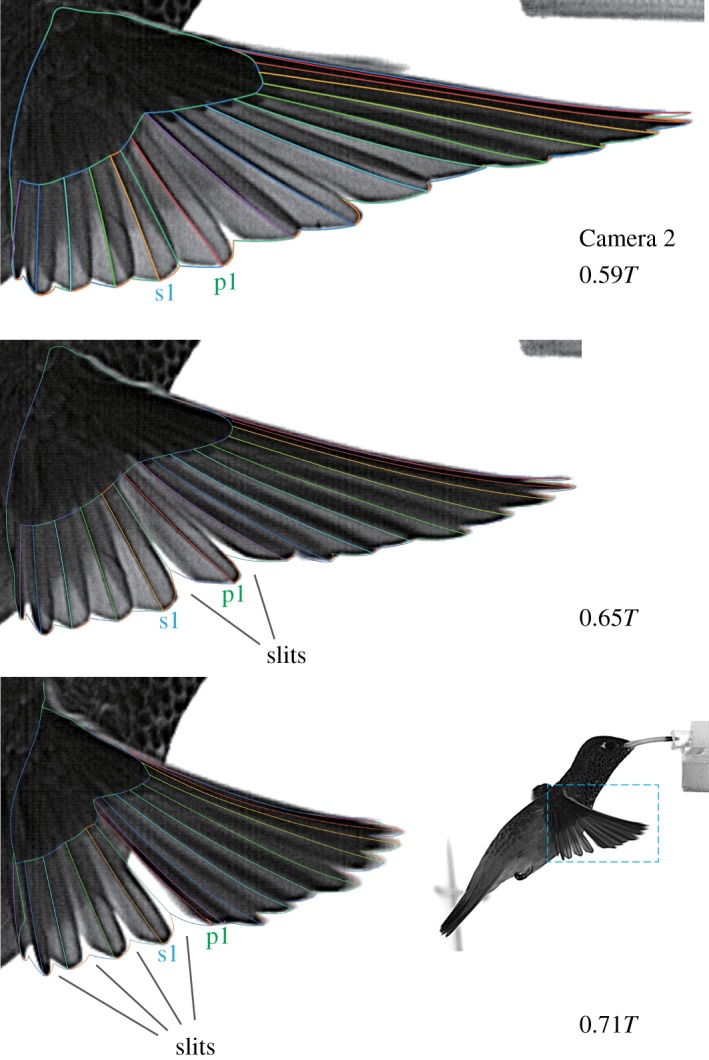
Several slits were observed during upstroke.

The local twist gradients Δ*θ*/Δ*L*_SP_ for the wing sections generally show negative values during downstroke and positive values during upstroke (figure 15*a*). The proximal (20% and 40% *L*_SP_) sections reached the minimum values at early downstroke (−1.00^°^ mm^−1^ for 20% *L*_SP_ section and −0.73^°^ mm^−1^ for 40% at 0.077*T*), whereas the distal sections gradually decreased until mid-downstroke, but overall, the values were kept relatively constant at approximately −1^°^ mm^−1^ for most of the downstroke period. At supination, the signs for all the sections changed from negative to positive almost simultaneously. In contrast to downstroke, during upstroke, a remarkably large local twist gradient was observed for the 20% *L*_SP_ section (blue triangles in figure 15*a*), while the other locations kept a similar amplitude as the downstroke (approx. 1^°^ mm^−1^, except for the 1.6^°^ mm^−1^ for the 40% *L*_SP_ section at 0.61*T*). The maximum local twist gradient for the 20% *L*_SP_ section reached 3.15^°^ mm^−1^ at 0.65*T*. This contrasting pattern between downstroke and upstroke is even clearer by examining the spanwise variations of the local twist gradient for two representative time frames at downstroke (0.25*T*) and at upstroke (0.65*T*) (figure 15*b*, green open circles and purple filled circles, respectively; the timings are indicated by the vertical bands in figure 15*a*). At 0.25*T*, the local twist gradient was nearly uniform across the wing long axis, whereas at 0.65*T*, a substantially large twist occurred at the proximal (20–30% *L*_SP_) sections. Coincidentally, a few slits (gaps) were observed among the secondary feathers, and particularly between the first secondary (s1) and the first primary (p1), at approximately the same timing during upstroke (0.59*T*–0.71*T*, figure 16). The slits were observed for all four recorded sequences and hence not a rare event, although it is unknown whether this is a common feature among other individuals.

### 3.9. Kinematic angle of attack

**Figure 17.**
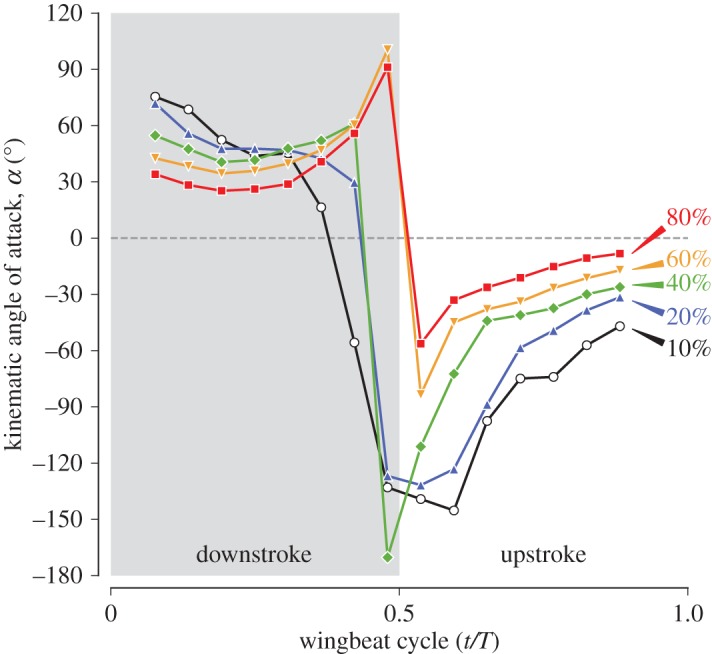
Kinematic angles of attack. Grey shaded region indicates the downstroke period.

The kinematic angles of attack (AoAs) *α* for the wing sections are shown in figure 17. In general, the AoA was positive during downstroke and negative during upstroke. Reversals of the AoAs occurred near pronation and supination, but the detailed reversal timings were different from those of the AoIs. The AoIs for all the wing sections changed their signs simultaneously between 0.42*T* and 0.48*T* (figure 14*a*), and the reversals of the AoAs for the proximal sections (10, 20 and 40% *L*_SP_) occurred at similar timings, but the reversals of the AoAs for the distal sections (60% and 80% *L*_SP_) occurred between 0.48*T* and 0.54*T*.

During mid-downstroke, the angles of attack for the distal sections (at 0.25*T*, 35^°^ and 26^°^ for the 60% and 80% *L*_SP_ sections, respectively) were smaller than those for the proximal sections (at 0.25*T*, 48^°^ and 41^°^ for the 20% and 40% *L*_SP_ sections, respectively), but they were still relatively high values. Note that these values were already achieved at early downstroke and remained relatively constant. By contrast, the AoIs exhibited more sinusoidal waveforms (figure 14*a*). Towards the end of downstroke, the distal sections increased their angles of attack. Specifically, the 80% *L*_SP_ section became nearly perpendicular to the relative wind (92.9^°^ at 0.48*T*).

By contrast, during upstroke, the angles of attack took higher magnitudes at the onset, but then the magnitudes gradually decreased towards the late upstroke, and no wing section became perpendicular to the relative wind. During mid-upstroke, the magnitudes were similar to those during mid-downstroke for the proximal sections (at 0.77*T*, −47^°^ and −35^°^ for the 20% and 40% *L*_SP_ sections, respectively), but the magnitudes were smaller for the distal sections (at 0.77*T*, −25^°^ and −14^°^ for the 60% and 80% *L*_SP_ sections, respectively). This asymmetry in the AoA for half-strokes is likely to be contributing to the aerodynamic vertical force asymmetry.

## 4. Discussion

Our study demonstrated the effectiveness of the proposed method, and provided direct evidence that the hummingbird wing dynamically changes its shape during hovering flight, and the details of such dynamic wing morphing for a hummingbird were quantified. To summarize, the wing area increased during downstroke and decreased during upstroke; the maximum area was 18% larger than the minimum area; the wing was bent in both the in-stroke-plane direction (maximum 13^°^ at mid-downstroke and minimum −30^°^ at early upstroke for the wing tip) and out-of-stroke-plane direction (two positive peaks: 11^°^ at mid-downstroke and 12^°^ at early upstroke for the wing tip); the wing had camber such that it had an upward convex cross-sectional shape in both strokes (half-stroke averages for the 60% *L*_SP_ section are 8.2^°^ and −6.3^°^ for downstroke and upstroke, respectively); and the wing had the spanwise twist for both strokes in a manner such that the kinematic angles of attack for the wing distal sections were smaller than those for the wing proximal sections.

### 4.1. Potential causes and aerodynamic implications of dynamic wing morphing

We will discuss the potential causes for each wing morphing described above, and we will also predict the impact of these morphings on the aerodynamic force. Note that the interaction between the shape of a flexible object and the aerodynamic force and moment (torque) is not that simple in reality, because any change in shape alters the flow field and thus the aerodynamic force, which again alters the shape, and this process repeats. This phenomenon, the so-called aeroelasticity or in general fluid–structure interaction (FSI), is beyond the scope of this study. Therefore, care should be taken when reading the descriptions below, where we discuss as if the wing is statically deformed first and then the aerodynamic force is generated, which is not precise but rather a more qualitative estimation.

#### 4.1.1. Wing area

As expected, the wing length was nearly constant (figure 7) but with a remarkable 18% variation in the wing surface area (table 2 and figure 8). The overall trend of the RTD variation for the entire wing (‘total’ in figure 8*b*) was similar to the trend of the total wing area (‘total’ in figure 8*a*). This result indicates that the change in wing area is arising from the sliding (fanning) motion of the feathers. This is especially evident for the secondary feathers, where the wing area ratio was the largest in the secondary region (*A*_W,0.42*T*_/*A*_*W*,0.94*T*_=1.40 in table 2), which corresponds to the largest RTD variation for the secondary feathers (figure 8*b*, blue crosses).

What causes the RTD variation? The fact both wing area and RTD decrease throughout the supination indicates that the contribution from aerodynamic force (which should peak at the mid-strokes) is minor. However, the large variation in secondary feathers implies that the inertial force due to wing strokes alone would not explain all the sliding motion. The adjustment of individual feather direction at the root of each feather (or set of feathers) through muscle actuation may be included.

Note that the overall trend in wing area variation is consistent with the aerodynamic vertical force (weight support) found in previous studies [5,6]. Therefore, the asymmetry in the wing area is likely to be contributing to the asymmetry in the aerodynamic force between half strokes. However, the maximum wing area during downstroke was only 18% larger than the minimum wing area during upstroke, and the downstroke-average wing area (1424 mm^2^) was only 10% larger than the upstroke-average wing area (1299 mm^2^). Therefore, other factors, such as AoA, camber or relative wind speed difference, would also be included for the asymmetrical aerodynamic force generation during downstroke and upstroke.

#### 4.1.2. Spanwise bending

The wrist joint is probably located near the leading edge of the 10% or 20% *L*_SP_ sections (see Methods). Therefore, the large local bending gradients that were observed at the proximal wing locations (blue triangles and green diamonds in figure 10*c* and *f*) imply that the majority of the bending is caused at the wrist joint, although the contributions of the bending of individual primary feathers are also apparent, particularly in the out-of-stroke-plane direction (orange inverted triangles and red squares in figure 10*f*).

The phase difference in spanwise bending in which the proximal portion of the wing is advanced to the distal portion for both in- and out-of-stroke-plane directions (figure 10*a*,*c*) indicates passive deformation of the wing due to either inertial or aerodynamic force. The aerodynamic force vector for a single wing during downstroke would be perpendicular upward to the stroke plane with backward and lateral tilts. During the first quarter of the downstroke (0<*t*<0.25*T*), the lateral tilt for the right wing would be pointing to the left, whereas during the second quarter (0.25<*t*<0.5*T*), it would be pointing to the right. The component perpendicular to the stroke plane roughly corresponds to the lift, whereas the sum of the other components roughly corresponds to the drag. They may be responsible for the backward bending and upward bending for the in-stroke-plane and out-of-stroke-plane directions, respectively, especially during mid-downstroke. Similarly, during upstroke, the aerodynamic force vector would be pointing perpendicular upwards (lift) with forward and lateral tilts (drag). They are again in agreement with the directions of the bending components, but the quick negative peak of the in-stroke-plane bending just after supination (figure 10*b*) cannot be attributed to the aerodynamic force because the relative wind speed is nearly zero at this time instance (see electronic supplementary material, figure S9); hence, the aerodynamic force would be very small. Therefore, the large in-stroke-plane bending during supination and early upstroke is probably due to the inertial force. Neuromuscular actuation may also be involved.

The impact of 10–15^°^ bending (figure 10*b*,*e*) on the aerodynamic force generation is unclear at this moment and awaits a direct comparison with, e.g. the blade-element method or CFD. The out-of-stroke-plane bending may be contributing to the rolling (lateral) stability, i.e. preventing the bird from banking to the left or right by small perturbations or gusts, similar to the coning angle (effective dihedral) in a helicopter. However, one needs to be careful when seeking for such a stability study because, in reality, the wing deformation itself can be affected by the wind if it is strong. To determine the stability against abrupt wind (gust), an experiment using a real bird, a robotic model or a numerical simulation with FSI would therefore be necessary. By contrast, the aerodynamic function of the in-stroke-plane bending is less obvious. It may be the by-product for achieving the sinusoidal waveform in the positional angle for the wing distal portion (figure 10*a*, filled black circles).

If the aerodynamic impacts are small, then it is even possible that the spanwise bending is in reality not particularly desired or favoured by hummingbirds for aerodynamic benefits but rather an inevitable consequence when using the lightweight structure (feathers rather than bones) to minimize the inertial power. Any bending results in a decrease in wing length, which is generally unfavourable for efficient aerodynamic force generation.

#### 4.1.3. Camber

The camber was overall positive during downstroke and negative during upstroke (figure 13). However, the wing is flipped around the long axis during upstroke (figures 6 and 14*a*); thus, the bulging side of the wing is pointing upwards during both strokes. This would be beneficial in efficient lift generation, most of which will be used as the upward force because of the nearly horizontal stroke plane.

The mechanism behind camber generation cannot be determined from the present study alone, but it is likely to be similar to the automatic (passive) formation of the camber in insect wings by Ennos [14], where he in fact mentioned the wings of birds and hummingbirds in particular. The primary feathers of the hummingbird wing are attached to the hand bone with some swept angle, and the hand bone forms part of the leading edge, which undergoes torsional rotation [40]. These are consistent with the automatic camber generation mechanism for an insect wing. Furthermore, it was explained that the backward curve (curve towards the trailing edge) of the ‘spars’ (corresponding to rachides in the present case) and non-parallel, diverging-manner branching of the spars from the leading edge both cause extra cambers, which are indeed observed in the hummingbird wing. The potential causes of the torsional rotation will be discussed later, but in addition to the rotation of the bones, direct forces (inertial or aerodynamic) acting on the feathers themselves might also have some roles. Immediately after the stroke reversal (0.019*T* or 0.54*T*), the large camber is already apparent, and the camber value is relatively maintained during the half-stroke. This result indicates that the aerodynamic force cannot be the major factor because the aerodynamic force would be nearly zero at the stroke reversals. However, the aerodynamic force may be helping the wing to maintain camber during mid-strokes. It would be possible that more complicated mechanisms such as fluid–structure interaction (FSI) or tribology (i.e. friction between adjacent feathers) are involved.

The magnitudes of the half-stroke average camber |*h*_max_/*c*| at wing sections were slightly larger during downstroke compared to those during upstroke (table 4), which may also be contributing to the vertical upward force asymmetry found in the previous studies. The extent of the camber (table 4) is comparable to that found in hovering hoverflies, where they showed positive (approx. 0 to 10%) camber during downstroke and negative (approx. −10 to 0%) camber during upstroke (fig. 9c,f in [41]). A study on the wing of a barn owl in forward flight showed that the maximum camber is nearly 15%, which occurs at the beginning of upstroke, while the minimum camber is somewhere around −5 to 0% during upstroke [28]. Both the extent and the timing are quite different from those in the present hummingbird wing in hovering, but it would be interesting if a hummingbird wing in forward flight shows a similar tendency as that of the owl or if the trend is instead unchanged from hovering. If the former is the case, then it indicates that the temporal camber variation found in the owl is generally favourable for forward flight, and the hummingbird wing has the adaptability to exhibit different morphings in hovering and forward flight. If the latter is the case, then further consideration is needed, i.e. there is more than one explanation: either the hummingbird wing is more ‘tuned’ to hovering than forward flight, or the hummingbird wing is suboptimally designed between the demands from hovering and forward flight. Alternatively, the favourable dynamic camber for an unfoldable wing may be different from that for the foldable (conventional) bird wings. Further studies are needed to answer these questions.

#### 4.1.4. Spanwise twist

We found a washout twist for both half-strokes (figure 14*b*). This result is similar to what was found in hovering hoverflies [41], but the details are slightly different. In the hummingbird wing, the magnitude of the spanwise twist |Δ*θ*| for each wing section was substantially larger during downstroke than that during upstroke (figure 14*b*). This is because the angle of incidence (AoI) for the 10% *L*_SP_ section was maintained nearly constant throughout upstroke (at approx. 100^°^), whereas during downstroke, the AoI for the same section followed a sinusoidal trend as the other sections did (black open circles in figure 14*a*). As the 10–20% *L*_SP_ sections roughly coincide with the wrist joint (see Methods), this result indicates that the hand bone rotation at the wrist joint is related to the torsional rotation during upstroke. In fact, this agrees with the findings in the stereo X-ray kinematic measurement for ruby-throated hummingbirds [40]. This torsional rotation of the wrist joint might cause the large peak of the local twist gradient at 0.65*T* (figure 15*a*, purple band) and the slit between the secondary and primary feathers (figure 16).

During upstroke, the maximum AoIs were reached earlier for the more distal sections than for the intermediate or proximal sections, i.e. the 80% and 60% sections reached the peak at 0.59*T*, the 40% section at 0.65*T*, the 20% section at 0.71*T* and finally, the 10% section at 0.82*T* (figure 14*a*). This result appears to be quite similar to the so-called torsion wave (or torsional wave) mentioned in the insect wing studies [13,41–43]. According to these previous studies, the fact that this torsion wave propagates from tip to base indicates that a passive mechanism, either aerodynamic or inertial, is involved. Hedrick *et al*. [40] also mentioned that the movement of the fourth primary could not be explained by the motion of the bones. According to a recent study on the hummingbird wing model, the inertial torque is highly likely to be the major cause of the wing torsional reversal [44]. We believe (as briefly mentioned in the paper [44]) that the direction and shape of the feathers may be important. The wing torsional axis would roughly coincide with the shortest path, and each feather shaft is directed such that each of them places its centre of mass (CoM) behind the torsional axis. Specifically, the secondary feathers are aligned nearly perpendicular to the shortest path, and the primary feathers in the inner-primary region are aligned obliquely with the shortest path; therefore, the CoMs are naturally located behind the torsional axis. However, the primary feathers in the outer-primary region are aligned nearly parallel to the shortest path, but they have substantial natural curvature towards the wing trailing edge (even without external force), which also would result in their CoMs locating behind the torsional axis. Similarly, the mid-primary feathers have moderate natural curvature and moderately oblique orientation.

The torsional reversal at supination appears to be ‘advanced’ to the stroke (positional) reversal (figure 14*a*), but this is because we chose the wing tip for defining the downstroke period and thus stroke reversal timing. If one uses the motion of the wing proximal portion (e.g. wrist joint, which should be close to the *P*_10_ or *P*_20_) for defining the stroke reversals, the torsional reversal would be occurring in the middle of supination (figure 10). This is in fact similar to what was found in the numerical simulation of a hawkmoth considering wing flexibility [45], where the phases of positional angle and feathering angle (corresponding to the AoI) in a flexible wing model were advanced to those in a rigid wing model.

#### 4.1.5. Angle of attack

Large magnitudes of the kinematic AoA across wing sections were observed during both half-strokes (approx. 25–50^°^ at mid-downstroke and −75 to −20^°^ at mid-upstroke; see figure 17). Together with the thin leading edge at the wing distal portion (because the p10 feather vane is constituting the leading edge there, figure 12), it is highly likely the LEV is generated during both mid-strokes on the distal portion of the wing, as observed in previous studies [5,6,46].

Young *et al*. [16] and Zheng *et al*. [17] conducted numerical simulations for a desert locust and a butterfly, respectively, in forward flight using CFD, where they compared different fidelity wings to assess the effect of dynamic wing morphing. They found that removing spanwise twist substantially deteriorates the aerodynamic force production efficiency (defined as the amount of vertical force per unit of aerodynamic power). A 15% reduction in the desert locust and a 30–50% reduction in the butterfly were found. In both cases, they found the smaller AoI at the wing distal sections than at the wing proximal sections, i.e. washout (for butterfly, washout during downstroke but washin during upstroke; however, a positive upward force was generated only during downstroke). This indicates high efficiency in the current hummingbird wing with a similar washout twist for both strokes (figure 14).

However, care must be taken because the lack of oncoming airflow resulted in a difference in the angles of attack. Our results show that the distal sections have smaller-magnitude kinematic angles of attack (AoAs) than the proximal sections, reflecting the spanwise twist, but this is different from the nearly uniform AoA along the wing long axis found in the above-mentioned desert locus in forward flight (fig. 6b,f in [38]). Rather, it is more similar to the AoA in a slow-flying pied flycatcher (fig. 2d in [7]), where the wind speed was merely 1 m/s. In their study, the downstroke average AoA was approximately 60^°^ at the proximal wing section and approximately 30^°^ near the wing tip, which are quantitatively close to the AoAs at mid-downstroke in the present study (figure 17). Note, however, that the ‘effective’ AoA in [7] is in reality not considering induced velocity (see also [4]) but only considering the wing kinematics (for their definition, refer to the supporting online material of another paper by the same authors: [37]). It is therefore equivalent to the ‘kinematic’ AoA in this study. They argue that the lower AoA in the distal section helps prevent the bursting of the LEV. It would therefore be reasonable to expect that the hummingbird wing twist may also be affecting the stability of the LEV and hence improving the aerodynamic force production efficiency.

Tobalske *et al*. reported the angles of attack for rufous hummingbirds, which are approximately 25 and −25^°^ for mid-downstroke and mid-upstroke, respectively (fig. 8C in [4], see 0 m s^−1^). They took the wing chord as ‘a line connecting the wrist and the distal tip of the 1st secondary’, which roughly corresponds to the 10% or 20% *L*_SP_ section in the present study. However, the magnitudes of the AoA for the proximal sections at mid-strokes were more than 30^°^ in this study. This discrepancy may simply be due to the difference in species, but it could also be due to the difference in the definitions of the AoA. They incorporated the induced velocity; hence, their angle is the so-called ’effective’ AoA in aerospace engineering. Conversely, we did not consider the induced velocity; thus, we call the angle the ‘kinematic’ AoA. Normally, the induced velocity has the downward vector component, thereby reducing the effective AoA compared with the kinematic AoA. Another possibility is that the higher AoA was for compensating the lower angular velocity. Tobalske *et al*. [4] reported a half-stroke average angular velocity (i.e. simply dividing the wingbeat amplitude by the half-stroke period) of approximately 180 rad s^−1^ for downstroke and approximately 160 rad s^−1^ for upstroke. The corresponding values for the present amazilia hummingbird were approximately 103 rad s^−1^ for both strokes.

Nakata & Liu [45] found in a hovering hawkmoth that the washout twist enhances aerodynamic efficiency because a twisted wing can produce a more straight downward flow (downwash) compared with a slanted downwash in a non-twisted wing (see fig. 4a(ii) and b(ii), and table S3 of [45]). A similar mechanism may be present in hovering hummingbird wings.

### 4.2. Limitations of the current study and potential directions

Our study provided good spatial resolution morphological data (tracking of all the rachides) for a live animal wing in flight with adequate temporal resolution (17 time frames for a wingbeat cycle). No physical contact with the bird was made; hence, undisturbed flight behaviour was observed. However, there were some drawbacks.

Even though the analysed stroke was based on the careful preliminary study (electronic supplementary material, figure S2), the current study treated only a single stroke cycle, and the lack of different individual data is regrettable. Therefore, care must be taken in interpreting the results. The dynamic wing morphing we observed and quantified is not necessarily representing the dynamic wing morphing of the hummingbirds in general. If one wants to seek the variation in wing morphing across different flight modes, individuals or species, it would be necessary to obtain the dynamic wing morphing from more than a single wingbeat. For such comparative studies, automatic shape reconstruction techniques would be helpful in drastically reducing the data acquisition time [15,28,36,47,48].

The information inside of the covert region is absent in the current method, which would be more important in forward flight, where the relative wind speed is considerably higher at the wing proximal sections compared to hovering. For a forward flight, accurate body shapes would also be necessary [49]. The tail shape and kinematics may also be of importance [50–53] but could be negligible for low-speed flights [54].

The surface roughness of the entire wing [30,31,35], which is based upon the individual feather shapes, and the small holes on feather vanes (porosity, air transmissivity or air permeability) [55,56] are known to affect the boundary layer and thus aerodynamic force generation. However, none of these factors have been tested during flapping flight under dynamic wing morphing and would therefore be worth tackling.

The individual feather is probably experiencing the ‘rachis wise’ twist and bending, contributing to the dynamic morphing of the entire wing. Moreover, such individual feather deformation would not be limited to the hummingbird’s wing. The separated outer primary feathers in large landbirds appear to be showing such twist and bending [57]. It might be possible that individual feathers contain the intrinsic function to automatically adjust their shapes to achieve a favourable flow field via e.g. suppressing the burst of the LEV on each feather. The physical contact between the adjacent feathers may also contribute to maintaining wing shape, but the force transmission may be primarily restricted to the dorsoventral (perpendicular to the wing/feather surface) direction, in contrast to the membranous wings where the forces would also be transmitted to the tangential direction (parallel to the wing/feather surface) via tension. Therefore, single and multiple feather experiments or numerical simulations, preferably considering FSIs, are strongly encouraged for further elucidation of the function of the feathered wing. For an experiment focusing on the individual feather kinematics/deformation, an even higher spatial and temporal resolution tracing than that in the current study will be required. We did not show the wing area and the RTD for individual feathers because such data were too noisy to observe the trends. Summation over the region or over the entire wing reduced the noise.

The three-dimensional dynamic wing morphing of living organisms may be inspirational in designing the wings for flapping-wing micro air vehicles (MAVs) [58]. For instance, in a series of a membranous MAV wing-flapping experiments, it was found that both the aerodynamic vertical force and the mechanical efficiency increase as the wing has the more similar outline to that of the hummingbird or the wing membrane is loosened to allow spanwise twist [59].

## Supplementary Material

Supplementary materials PDF from Quantifying the dynamic wing morphing of hovering hummingbird

## Supplementary Material

Reconstructed 3D hummingbird wing grid data

## Data accessibility

Videos of the hovering hummingbird and the reconstructed three-dimensional wing grid can be accessed via the electronic supplementary material.
