# Supplementary material for: Quantifying the dynamic wing morphing of hovering hummingbird
Source: R Soc Open Sci. 2017 Sep 20;4(9):170307. doi: 10.1098/rsos.170307 (PMC5627076; doi:10.1098/rsos.170307)
Supplement: Supplementary materials PDF from Quantifying the dynamic wing morphing of hovering hummingbird [file rsos170307supp1.pdf]

Electronic supplementary materials

Article title: Quantifying the dynamic wing deformation of hovering hummingbird

Authors: Masateru Maeda, Toshiyuki Nakata, Ikuo Kitamura, Hiroto Tanaka, and Hao Liu

Journal title: Royal Society Open Science

Supplementary videos

Videos S1–S4: high-speed videos of a hovering hummingbird

The videos show the 69 time frames recorded by the four synchronised high-speed digital video cameras. The videos were recorded at 2000 frames per second (fps) and played at 30 fps; therefore, the playback speed is 0.015 (approximately 1/67) times the real speed. The videos were originally stored as the MRAW file type (12 bit grey scale), which were then exported as the AVI file (8 bit grey scale) using the Photron FASTCAM Viewer.

Video S5: hummingbird hovering video with traced lines

The video is created from the screenshot of the CAD (Rhinoceros 5) files consists of 17 time frames, where the red and blue lines are the wing outlines and boundaries whereas yellow lines are the rachides, as in figures 1A–E. The video is played at effectively 6 fps; therefore, the playback speed is 0.003 (approximately 1/333) times the real speed.

Video S6: reconstructed hummingbird wing model video

The video shows the top and lateral views of the reconstructed wing model for a single wingbeat cycle (17 time frames). Shortest path (magenta) and wing cross sections (black, 10% L_{SP}; blue, 20% L_{SP}; green, 40% L_{SP}; yellow, 60% L_{SP}; and red, 80% L_{SP}) are shown.

Supplementary methods

Calibration tool

The calibration tool and its dimensions are shown in figure S1.

Selection of the wingbeat period for detailed analysis

We wanted to obtain the wing surface information in addition to the wing outlines (edges), which is essential for accurately calculate wing surface area and camber. For that, sometimes markers are attached (or painted)

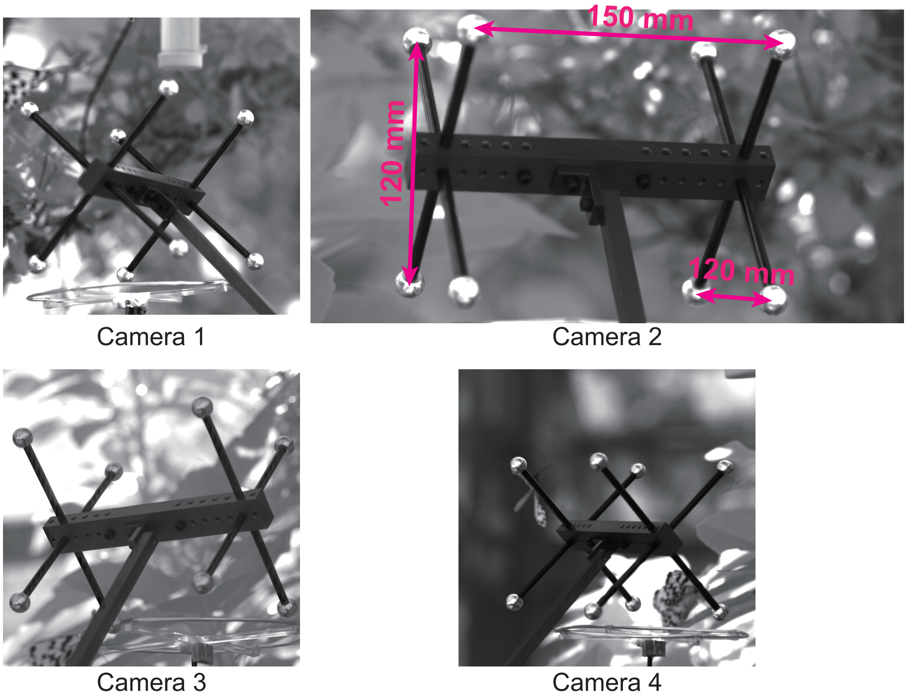

Figure S1: The calibration tool was made of eight metal spheres and connecting rods.

on the wing surface , whereas in some cases natural features or projected random dots are used. In the present study, the direct contact with the bird was prohibited by the zoo. We therefore decided to use the feather shafts (rachides or rachises) as the major feature on the wing surface. They are supplemented by the boundary between primary and secondary feathers, and the boundary between covert feathers and the other parts. These features are not sharp enough for the automatic reconstruction with the adequate accuracy, partly because of the motion blur due to the slow shutter speed (1/3000 s), which was inevitable because we dependent upon the natural lighting (the addition of any artificial lighting was prohibited). Therefore we needed to track the natural features manually, which required us to select limited number of image frames to be analysed.

Consequently, the wingbeat cycle for the detailed analysis was selected based on the preliminary analysis checking the wingbeat frequency for each stroke cycle. Four separate stable hover-drink sequences were analysed (figure S2) each consisting of 45, 26, 12, and 29 wingbeat cycles. Figure S2A shows the box plots and swarm plots for the image frames per cycle (dividing 2000 frames per seconds by this number yields the wingbeat frequency). Figure S2B-E) illustrate the time history of wingbeat frequency. It seems the bird tends to increase the wingbeat frequency when it ends drinking (except for the 4th sequence) but overall the frequency was within the range of 28-32 Hz. The first sequence (figure S2B) was the longest of them, and there was a five continuous wingbeats with the same frequency at 29.4 Hz before the end of drinking. We selected the middle of them for the further analysis for wing kinematics and deformation (annotated by orange texts in figure S2A and B; also highlighted by the green stripe in figure S2B). This is the median frequency of the 1st sequence and very close to the median frequency for the pooled data (29.9 Hz, figure S2A). We also tracked the wingbase and

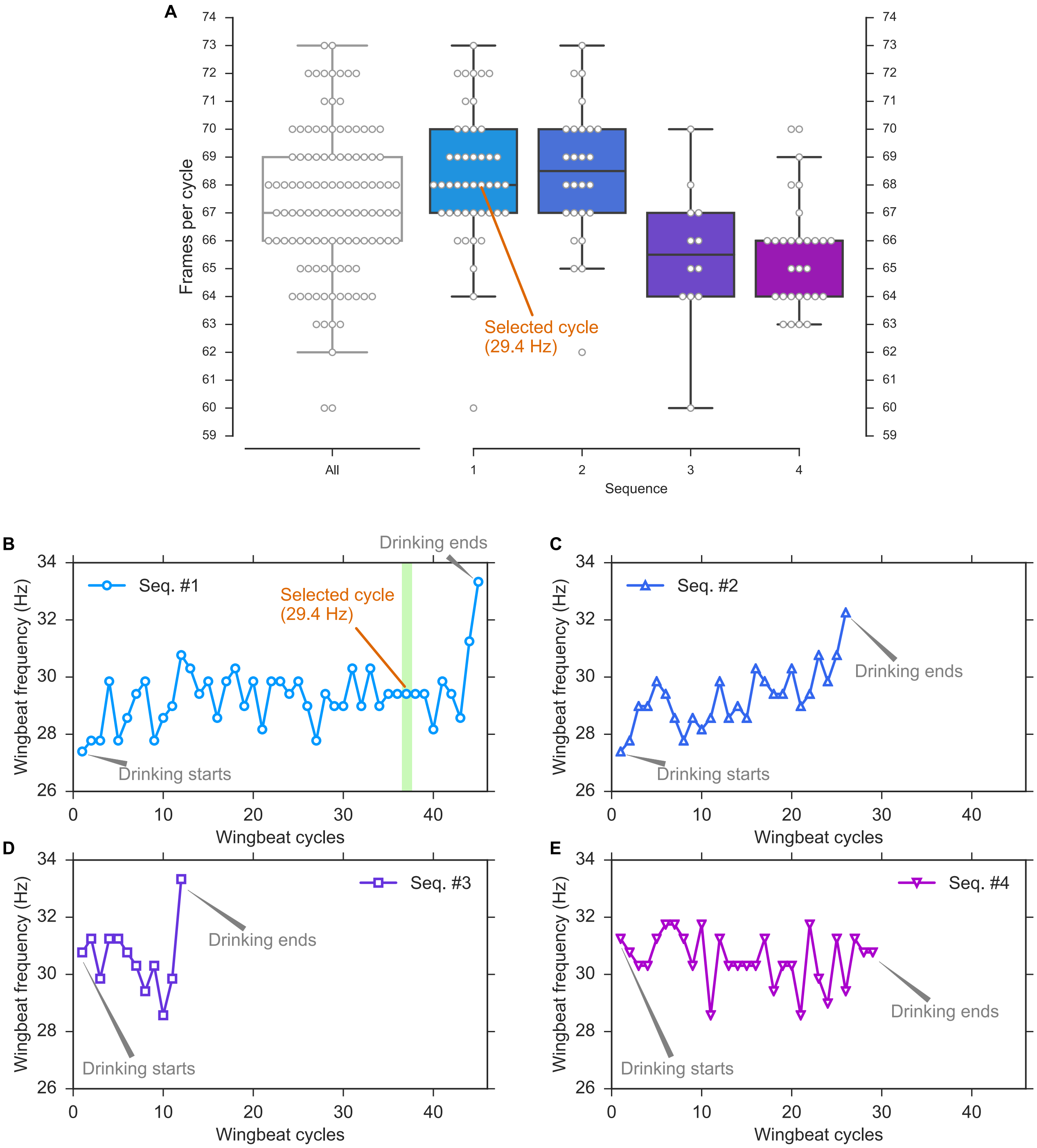

Figure S2: Wingbeat frequency from preliminary kinematic analysis. (A) Box and swarm plot for 1st, 2nd, 3rd, and 4th flight sequences, along with the pooled result. Note the vertical axis represents the image frames per wingbeat cycle. Since the frame rate was 2000 frames per second, dividing 2000 by the frames per cycle results in the wingbeat cycle in Hz, e.g. if a cycle composes of 69 frames, then the frequency is $2000/68 \simeq 29.41$ Hz. Therefore 59 frames corresponds to 33.9 Hz and 74 frames corresponds to 27.0 Hz. (B-E) Temporal variation of wingbeat frequency for 1st (B), 2nd (C), 3rd (D), and 4th (E) sequences. The wingbeat cycle around 37th cycle from the 1st sequence (B) was chosen for the further analysis.

wingtip for the stable cycles around the selected cycle (near the green stripe in S2A). The calculated wingbeat amplitudes were within 105 and 115 degrees. For the selected cycle, we did much more detailed analysis, which finally resulted in slightly different values mentioned above (28.8 Hz and 103 deg, Table 1).

Determination of the global coordinates

To calculate the stroke plane and stroke plane coordinates, the global coordinates need to be obtained beforehand.

The global Z axis (vertical upward) was obtained from the metallic ruler, therefore we have the horizontal plane. To obtain the other two axes, namely, backward (global X axis) and lateral right (global Y axis), we did the following. The left wingtip was tracked for four time frames ($t = 0.077T, 0.19T, 0.36T, 0.54T$). The vector connecting the left and right wingtips were averaged. Since the left wingtip was slightly lower than the right wingtip (the left-right wingtip vector was tilted from the horizontal plane by approximately 1.5 deg), we did not use this directly as the global Y axis. Instead, we called this left-right wingtip vector a Y' axis and assumed the yaw component of the Y' axis is correct but the roll component is not. Therefore, the global X axis was determined as the perpendicular direction to the Z and Y' axes. Next, the Y axis was determined as the perpendicular direction to the Z and X axes.

The cause of the slightly lower left wingtip compared to the right wingtip can be either one or combination of the followings: the error in the metallic ruler edge direction from the true gravitational acceleration vector, the calibration error of the left wingtip (since the left wingtip is nearly at the boundary of the calibration volume), or the slight asymmetry of the wing kinematics.

Line segments and triangle mesh

The line segments and the triangular elements (triangle mesh) are shown in figure S3 for the illustration purpose.

Shortest path length and wing length

The difference between shortest path (and its length) and the wing length are illustrated in figure S4. The theoretical minimum shortest-path length L_{SP} is achieved when the wing is totally flat, when the shortest-path length is equal to the wing length L_{W}. Otherwise, where there is any wing bending, the shortest-path length is greater than the wing length.

Selection of the stroke plane and stroke plane angle

The stroke plane (and therefore stroke plane angle) depends on the choice of the characteristic point used for the liner regression. If one choose another point than the wingtip, stroke plane would have been different. We checked this by using the points along the shortest-path in 5% L_{SP} spacings (figure S5). The results shows the stroke plane angles are within 2 deg variation (minimum 10.9 deg to maximum 12.9 deg). Therefore we chose wingtip for defining the stroke plane angle, which has been the standard for the previous literatures for flapping flight. Note here the stroke plane concept is connected to the flapping wing aerodynamic model, where wing

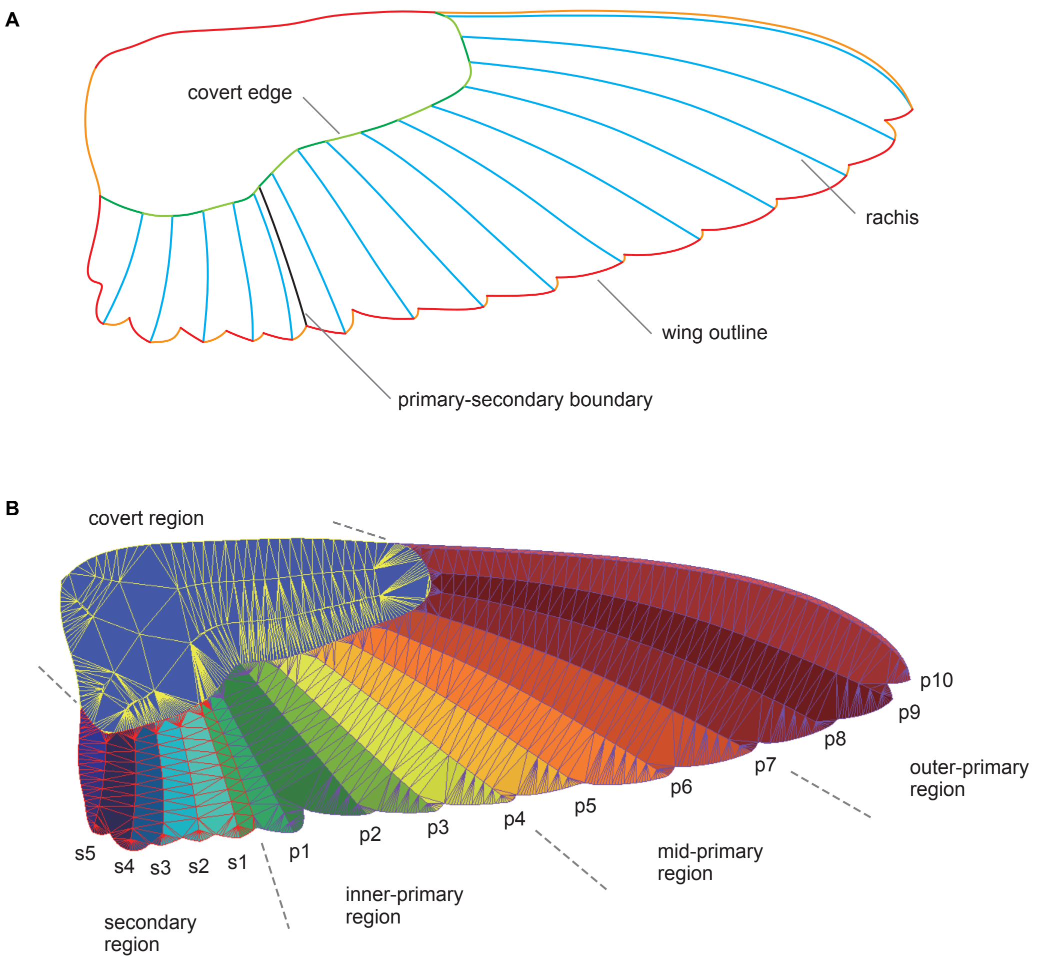

Figure S3: Example of the line segments (A) and triangle mesh (B). In A, wing outline is coloured by red and orange; rachides are coloured by light blue; the covert edge is coloured by dark green and light green; and the primary-secondary boundary is coloured by black (dual colours are for the illustration purpose to distinguish individual line segment). The wing area for each region and for the overall wing was calculated using the triangle mesh in B.

Section	$\overline{(h_{\mathrm{mid}}/c)}_{\mathrm{down}}$ (%)	$\overline{(h_{\mathrm{mid}}/c)}_{\mathrm{up}}$ (%)
10% L_{SP}	4.5	1.3
20% L_{SP}	7.3	-2.1
40% L_{SP}	9.9	-3.8
60% L_{SP}	7.7	-5.1
80% L_{SP}	5.0	-4.1

Table S1: Half-stroke averaged camber at mid-chord.

distal location usually generate major aerodynamic force, thus more likely representing the imaginary 'actuator disk'.

Supplementary results

Camber at mid-chord

The camber in the main text was defined as the maximum height h_{max} for each wing cross-section divided by the local chord length c. Here we also calculated the camber using the height at mid-chord (figure S6, table

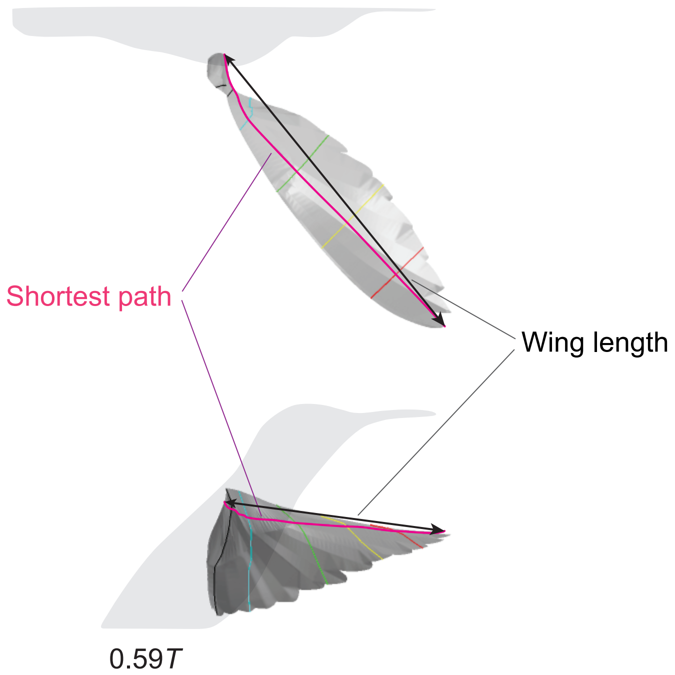

Figure S4: Shortest path length is the length along the shortest path (magenta curve), which is always greater than or equal to the and the wing length (black line with arrowheads).

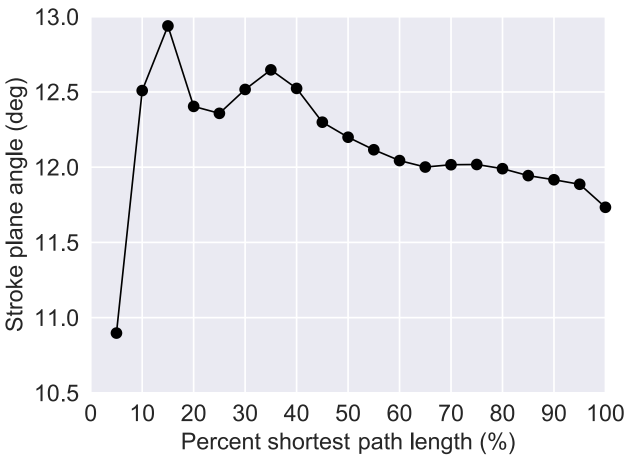

Figure S5: Stroke plane along shortest path.

S1). The mid-chord camber for the distal wing sections are similar to the maximum camber, indicating the chordwise location of the maximum camber is close to the mid-chord for the distal sections. However, the 10% section (black open circles) shows substantially smaller amplitudes for both half-strokes, indicating the maximum camber occurs somewhere else than the mid-chord. In fact, these are evident in the wing cross-sectional view in figure 12.

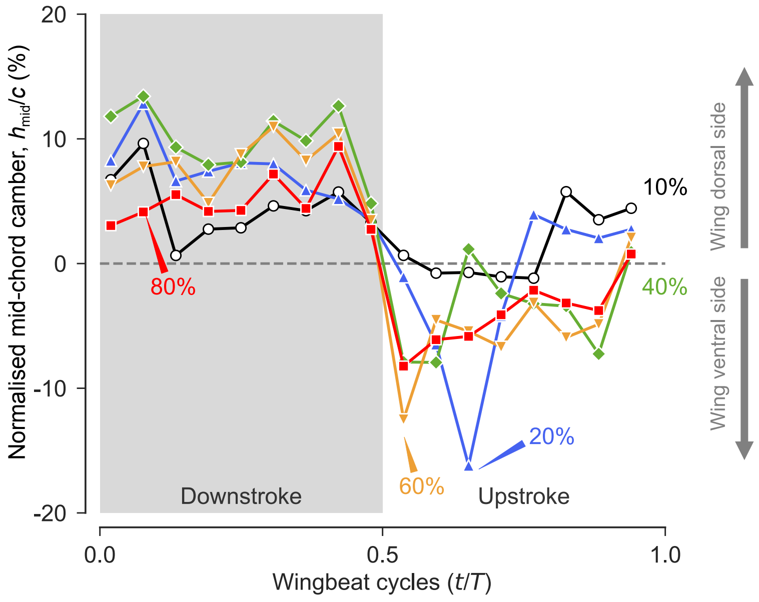

Figure S6: Instantaneous camber for the mid-chord. Gray shaded region indicates downstroke period.

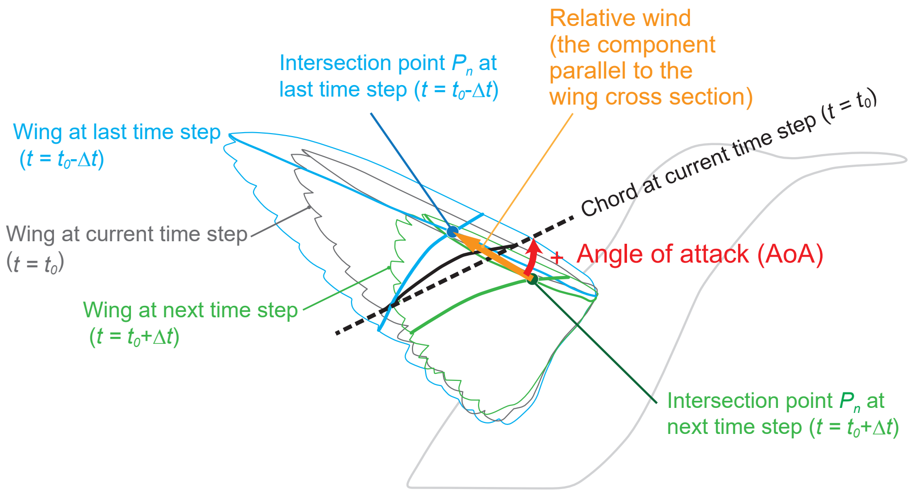

Figure S7: A detailed schematic for obtaining kinematic angle of attack.

Kinematic angle of attack and side-slip angle

The relative wind can have two components: one is the component parallel to the wing cross-section and the other is the components perpendicular to the wing cross-section. Most of the time the former is the major contributor and hence used for calculating kinematic angle of attack (AoA). The latter is sometimes referred to as the side-slip component and the angle based on the component and the wing chord may be called side-slip angle (SSA).

In figure S7 a detailed schematic for how to obtain AoA. A 3D relative wind vector (not shown) firstly obtained from the central difference from the P_n at last and next time steps (blue and green wings, respectively). Then the component parallel to the wing cross-section at current time step is extracted, and finally the AoA is calculated. Similarly, from the component perpendicular to the cross-section the SSA is calculated (not shown).

The time history of SSA is shown in figure S8. The SSA for the wing distal sections were kept either 0 deg

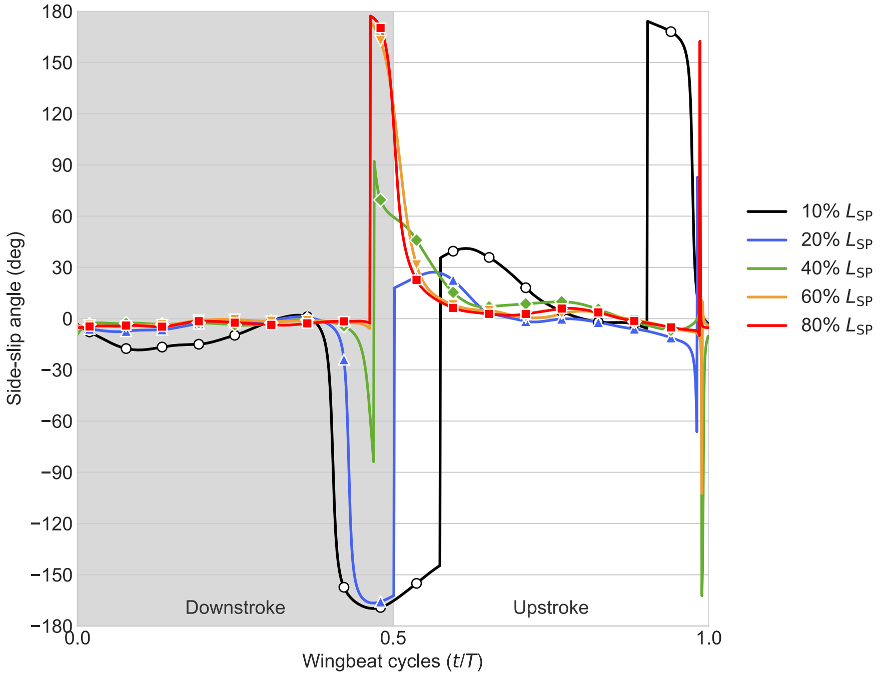

Figure S8: Side-slip angle (SSA). Gray shaded region indicates downstroke period.

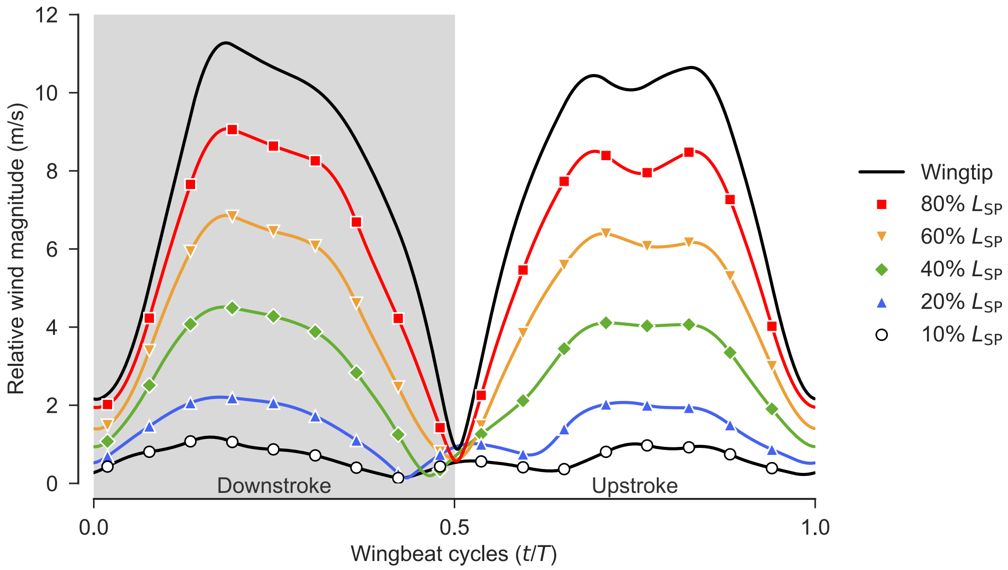

Figure S9: Relative wind speed. Gray shaded region indicates downstroke period.

or ± 180 deg for all the time except during stroke reversals (especially supination). Wing proximal sections, especially 10% section experienced the nonzero values at early half-strokes, but as we saw in S9 the relative wind speed (magnitude of the relative wind vector) itself is small for the current flight (hovering) and hence side force or the consequent aerodynamic power would not be large.

Relative wind speed and local chord length

Relative wind speed (magnitude of the relative wind vector for each wing cross-section) and the local chord length at wing sections are shown in figures S9 and S10. It is evident the wing distal sections experience much

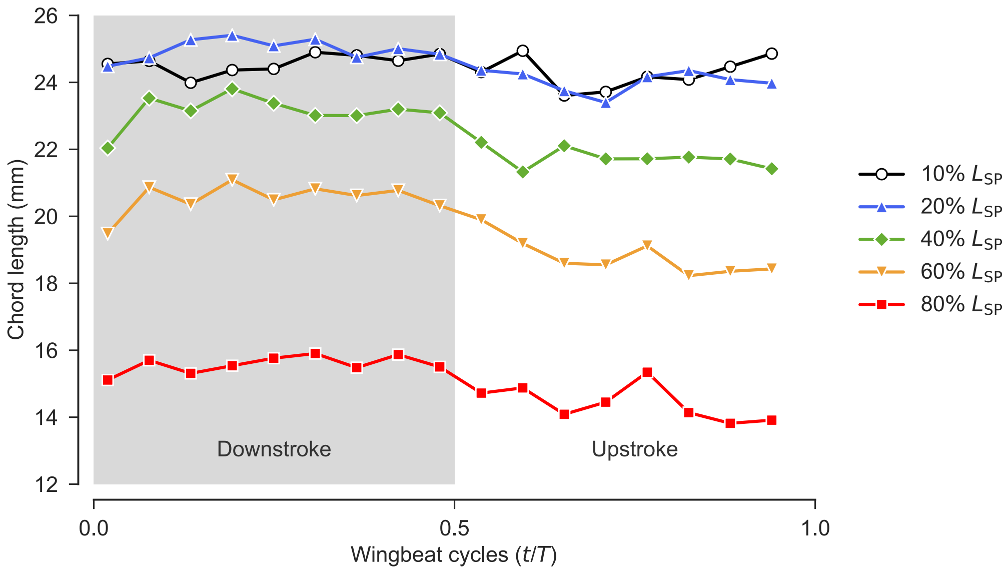

Figure S10: Local chord length. Gray shaded region indicates downstroke period.

higher relative wind speed than the proximal sections while having slightly shorter chord lengths. For example, the 60% section has roughly 80% chord length of the 20% section but the peak relative wind speed reached around four times larger. Since the amount of aerodynamic force is proportional to the square of the wind speed (and linearly proportional to the chord length), the wing distal sections would be the major contributor for the aerodynamic force. Note the difference in the angle of attack (AoA) and wing shape (camber) at different sections would surely affect the aerodynamic force coefficient and hence aerodynamic force, but that won't be enough to compensate the impact from the speed.
